# The Meiotic Recombination Activator PRDM9 Trimethylates Both H3K36 and H3K4 at Recombination Hotspots *In Vivo*

**DOI:** 10.1371/journal.pgen.1006146

**Published:** 2016-06-30

**Authors:** Natalie R. Powers, Emil D. Parvanov, Christopher L. Baker, Michael Walker, Petko M. Petkov, Kenneth Paigen

**Affiliations:** Center for Genome Dynamics, The Jackson Laboratory, Bar Harbor, Maine, United States of America; MRC Human Genetics Unit at the Institute of Genetics and Molecular Medicine at the University of Edinburgh, UNITED KINGDOM

## Abstract

In many mammals, including humans and mice, the zinc finger histone methyltransferase PRDM9 performs the first step in meiotic recombination by specifying the locations of hotspots, the sites of genetic recombination. PRDM9 binds to DNA at hotspots through its zinc finger domain and activates recombination by trimethylating histone H3K4 on adjacent nucleosomes through its PR/SET domain. Recently, the isolated PR/SET domain of PRDM9 was shown capable of also trimethylating H3K36 *in vitro*, raising the question of whether this reaction occurs *in vivo* during meiosis, and if so, what its function might be. Here, we show that full-length PRDM9 does trimethylate H3K36 *in vivo* in mouse spermatocytes. Levels of H3K4me3 and H3K36me3 are highly correlated at hotspots, but mutually exclusive elsewhere. *In vitro*, we find that although PRDM9 trimethylates H3K36 much more slowly than it does H3K4, PRDM9 is capable of placing both marks on the same histone molecules. In accord with these results, we also show that PRDM9 can trimethylate both K4 and K36 on the same nucleosomes *in vivo*, but the ratio of K4me3/K36me3 is much higher for the pair of nucleosomes adjacent to the PRDM9 binding site compared to the next pair further away. Importantly, H3K4me3/H3K36me3-double-positive nucleosomes occur only in regions of recombination: hotspots and the pseudoautosomal (PAR) region of the sex chromosomes. These double-positive nucleosomes are dramatically reduced when PRDM9 is absent, showing that this signature is PRDM9-dependent at hotspots; the residual double-positive nucleosomes most likely come from the PRDM9-independent PAR. These results, together with the fact that PRDM9 is the only known mammalian histone methyltransferase with both H3K4 and H3K36 trimethylation activity, suggest that trimethylation of H3K36 plays an important role in the recombination process. Given the known requirement of H3K36me3 for double strand break repair by homologous recombination in somatic cells, we suggest that it may play the same role in meiosis.

## Author Summary

Genetic recombination is the meiotic process by which novel combinations of alleles are passed on to the next generation. This process accelerates evolution by creating genetic diversity, and is also essential for successful meiosis. In mammals, the enzyme PRDM9 initiates recombination and determines the subset of sites within the genome—called recombination hotspots—that recombine. PRDM9 does this by binding DNA at hotspots and placing epigenetic marks. Previously, PRDM9 was only known to place the H3K4me3 mark at hotspots in living cells. Here, we show that PRDM9 places the H3K36me3 mark at hotspots, and that H3K4me3 and H3K36me3 coincide only at regions of recombination in germ cells. We prove that this coincidence is driven almost entirely by PRDM9; there is dramatically less coincidence between H3K4me3 and H3K36me3 when PRDM9 is absent. These results reveal a new enzymatic function for PRDM9, and a new epigenetic signature at hotspots that may restrict recombination to these sites. Since aberrant recombination can cause aneuploidy resulting in fetal loss, and abnormal genome rearrangements that underlie many congenital syndromes and some cancers, a thorough understanding of this fundamental process has potentially far-reaching implications.

## Introduction

Meiotic recombination, the process by which genetic material is exchanged between homologous chromosomes, occurs during gametogenesis in nearly all known sexually reproducing organisms. In addition to its role in creating genetic diversity, recombination ensures correct chromosome segregation during the first meiotic reductional division. Although many of the basic mechanisms of recombination are largely conserved from yeast to man, one aspect of recombination does differ greatly among taxa: namely, the molecular mechanisms specifying the genomic loci where recombination can initiate. In some organisms, such as *Drosophila melanogaster* and *Caenorhabditis elegans*, these loci show little or no DNA sequence specificity [1,2]. By contrast, in yeast and higher plants recombination is restricted to specialized sites, known as recombination hotspots, which are usually located in regions of open chromatin, such as promoters [3,4]. And finally, in mammals—including humans, chimpanzees, mice, cattle, and equids—the positions of recombination hotspots are determined by a specialized protein, the zinc finger-containing histone methyltransferase PRDM9 [5–9]. The fact that an intact *Prdm9* gene is absent from the genomes of lower taxa but found in the genomes of all mammals so far tested, with the exception of canids where it has become a pseudogene [10], suggests that determination of recombination hotspots by PRDM9 is a unique feature of mammalian gametogenesis.

PRDM9 protein is expressed exclusively in gametocytes during the leptotene to zygotene stages of meiotic prophase I [11], at which time it creates recombination hotspots by using its zinc finger array to bind at specific DNA sequences [12]. Genetic and molecular studies in humans and mice indicate that PRDM9 is likely the sole determining factor of recombination hotspot location [13,14]. PRDM9 then uses its PR/SET domain to locally trimethylate lysine 4 of histone H3 at adjacent nucleosomes [12–14]. We show here that it also trimethylates lysine 36 on the same nucleosomes. In normal meiosis, PRDM9 binds to DNA at hotspots and trimethylates the histones in the region; this causes the nucleosomes to move apart, creating a nucleosome depleted region and a configuration favorable for initiating DNA double-strand break (DSB) formation and DNA exchange between homologs [14]. The topoisomerase enzyme SPO11 is then recruited to catalyze a DSB at a subset of these PRDM9-activated hotspots [15,16]. It has been estimated that nearly 5000 hotspots in an average spermatocyte bind PRDM9 [14]. However, only several hundred of these acquire DSBs [17], all of which are repaired by homologous recombination repair (HRR), typically using the homologous chromosome as a template, although some may be repaired by sister chromatid exchanges [18]. A minority of DSBs goes on to form genetic crossovers; the remainder gives rise to non-crossovers with gene conversion. It should be noted that ‘recombination hotspots’ can be operationally defined as genomic regions that acquire PRDM9-dependent H3K4me3 marks or those that acquire meiotic DSBs. For the purposes of this paper, we will use the former definition, as it has been shown that the vast majority of DSB hotspots overlap the locations of PRDM9-dependent H3K4me3 hotspots [13].

Beyond specifying hotspot locations, PRDM9 is also necessary for successfully carrying out subsequent events in meiosis, as gametocytes in *Prdm9*-null mice are unable to repair the ectopic DSBs that are induced in the absence of PRDM9, undergoing meiotic arrest at an aberrant pachytene stage of Meiosis I, with complete failure of gametogenesis and sterility [11,13,19]. Importantly, in *Prdm9*-null mice, SPO11 catalyzes meiotic DSBs at sites such as promoters that are also marked by H3K4me3 [13]; however, proper DSB repair does not occur at these ectopic breaks, resulting in the observed meiotic failure and sterility [13,19]. It appears that, in addition to specifying the locations of hotspots, PRDM9 and/or its associated chromatin marks play an essential role in the repair of DSBs.

Recently, the isolated PR/SET domains of recombinant human and mouse PRDM9 were shown to have H3K36 as well as H3K4 trimethylation activity *in vitro* [20–22], making PRDM9 the only known mammalian histone methyltransferase capable of trimethylating both K4 and K36 [23], and suggesting a mechanism by which PRDM9’s methyltransferase activity and DSB metabolism might be functionally coupled. After PRDM9 dissociates from its binding site to create an opening for DSB formation, the covalently modified double-positive chromatin signature likely persists, providing a means for the SPO11 complex to discriminate between hotspots and other H3K4me3- and H3K36me3-enriched open chromatin sites in the genome. Additionally, H3K36me3 was recently shown to be necessary for homologous recombination repair (HRR) of DSBs in somatic cells [24,25], suggesting the possibility that PRDM9 is not only responsible for DSB placement, but also for its subsequent repair.

Here, we report that PRDM9 places both marks on the same nucleosomes *in vivo*, that the PRDM9 PR/SET domain is capable of placing both marks on the same histone molecule *in vitro*, and that the H3K4me3/H3K36me3-double-positive signature is unique to hotspots and the pseudoautosomal region (PAR) in germ cells. H3K36me3 does not coincide with H3K4me3 outside these regions in spermatocytes, and their coincidence at hotspots is entirely dependent on PRDM9. Collectively, these findings reveal a new enzymatic function for PRDM9 *in vivo*, and suggest a possible role for the H3K4me3/H3K36me3-double-positive signature in the initiation and repair of DSBs.

## Results

### Full-length PRDM9 trimethylates both H3K4 and H3K36 *in vitro*

Previous studies have shown that the isolated PR/SET domain from *PRDM9/Prdm9* (human and murine) expressed in E. coli (amino acids 195–385, 198–368, and 192–377, respectively) can trimethylate both H3K4 and H3K36 *in vitro* [20–22]. To determine whether this activity is a property of the full-length protein as well, we cloned full-length mouse *Prdm9*^Dom2^, expressed it in *E*. *coli* with an N-terminal MBP tag, and purified it to near homogeneity (S1 Fig). Testing the methyltransferase activity on histone peptide arrays confirmed that the purified full-length MBP-PRDM9 is able to methylate histone H3 at K4 and K36. PRDM9 showed methyltransferase activity when either of these residues was unmethylated, mono- or dimethylated (S2 Fig and S1 Table), indicating that, unlike many histone methyltransferases, PRDM9 is able to sequentially catalyze mono-, di-, and trimethylation of these histone H3 residues. However, PRDM9 showed the highest substrate specificity for di-methylated histone residues for both H3K4 and H3K36 (S3A Fig). In addition, we have confirmed and extended the finding by Wu et al. (2013) using the isolated PR/SET domain (murine, amino acids 198–368) [21], by showing that full-length PRDM9 is also able to trimethylate H3K9. Like H3K4 and H3K36, the highest substrate specificity of PRDM9 was towards di-methylated H3K9 (S3A Fig). The presence of other methylated residues in peptide H3 1–19 did not affect the methylation activity of PRDM9 at H3K4 or H3K9 residues (S1 Table). However, prior acetylation of H3K4, or H3K9 residues in peptide H3 1–19, and H3K36 residue in peptide 26–45, completely abolished the ability to methylate them (S3C Fig). Methylation was also prevented by prior phosphorylation of S10 and T11, presumably by preventing binding of the histone tail at the PRDM9 catalytic site (S3D and S3E Fig). The data also suggest that phosphorylation at T3 attenuates methylation (S3B Fig).

### PRDM9 PR/SET domain trimethylates H3K36 less efficiently than it does H3K4

**Fig 1:**
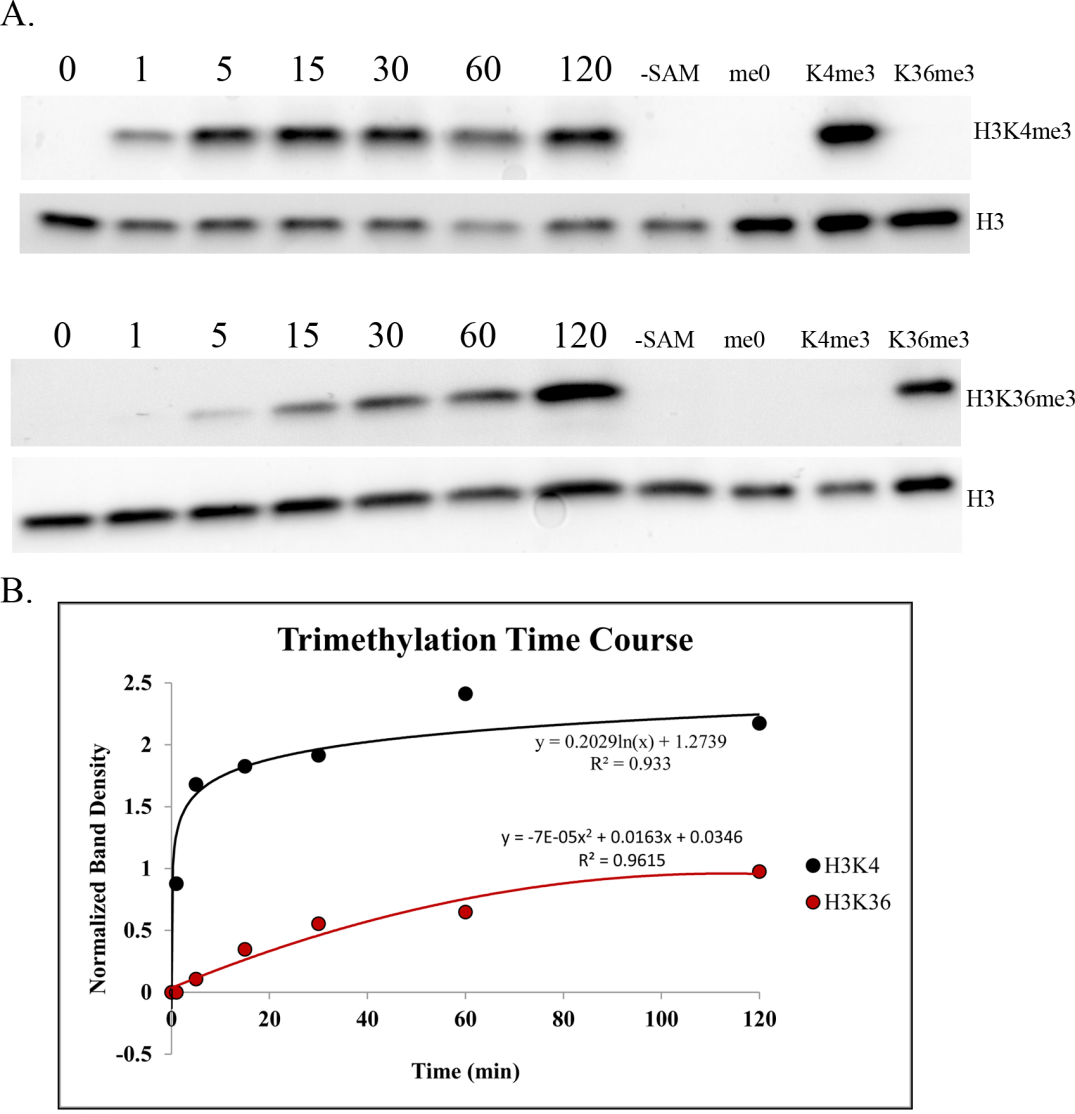
Intrinsic H3K4 and H3K36 trimethylation activities of the PRDM9 PR/SET domain. **(A)** Western blots of a typical histone methyltransferase assay time course for PRDM9 PR/SET domain, with unmodified recombinant histone H3 as substrate. Duplicate blots were probed with α-H3K4me3 and α-H3K36me3 antibody, respectively. Both blots were then stripped and re-probed with α-H3 antibody to determine the total amount of histone H3 in each lane. Numbers indicate time in minutes; “-SAM” indicates a negative control reaction that was run without the methyl donor S-adenosylmethionine; the last three lanes contain the denoted recombinant histone, to demonstrate antibody specificity. **(B)** Histone methyltransferase time course plot. The y-axis represents the ratio of the density of each H3K4me3 or H3K36me3 band to the corresponding H3 band, to control for the total amount of histone H3 in each lane.

We performed a kinetic histone methyltransferase assay using recombinant murine PRDM9 PR/SET domain and recombinant histone H3 as substrate to compare the relative efficiency of the two reactions, finding that, *in vitro*, the PRDM9 PR/SET domain trimethylates H3K36 much more slowly and less efficiently than H3K4 when unmodified histone H3 is the substrate (Fig 1). This result is consistent with our *in vivo* ChIP-seq data (below) and our histone peptide array data (S3A Fig), as well as with a previously published study of the human PRDM9 SET domain (amino acids 195–385) with histone H3 peptides as substrate *in vitro* [20], which showed the H3K36 mono- and dimethylation steps to be rate-limiting and considerably less efficient than any step of the H3K4 trimethylation reaction.

### PRDM9 trimethylates H3K36 at hotspot nucleosomes in spermatocytes

To determine whether PRDM9 trimethylates H3K36 at hotspots *in vivo*, we performed ChIP-seq with an anti-H3K36me3 antibody on 14 day post-partum (dpp) germ cells isolated from two mouse strains: C57BL/6J (B6) and B6.Cg-*Prdm9*^<tm1.1Kpgn^>/Kpgn (KI). The latter is a knock-in strain identical to B6 (*M*. *m*. *domesticus*) except for the last exon (exon 11) of *Prdm9*, which has been replaced with exon 11 of *Prdm9*^Cst^, the allele of *Prdm9* found in CAST/EiJ mice (*M*. *m*. *castaneus*) [14]. Exon 11 encodes *Prdm9*’s zinc finger array, so the DNA binding specificity of PRDM9 is the only genetic difference between the two strains. Because the *Prdm9*^Cst^ allele is highly divergent from the endogenous *Prdm9*^Dom2^ allele present in B6, the B6 and KI strains have very different hotspots [14]. For the purposes of this study, we define hotspots as strain-specific H3K4me3 peaks. As the two strains are genetically identical except for the DNA-binding domain of *Prdm9*, any H3K4me3 peaks not shared between the two strains are PRDM9-dependent.

**Fig 2:**
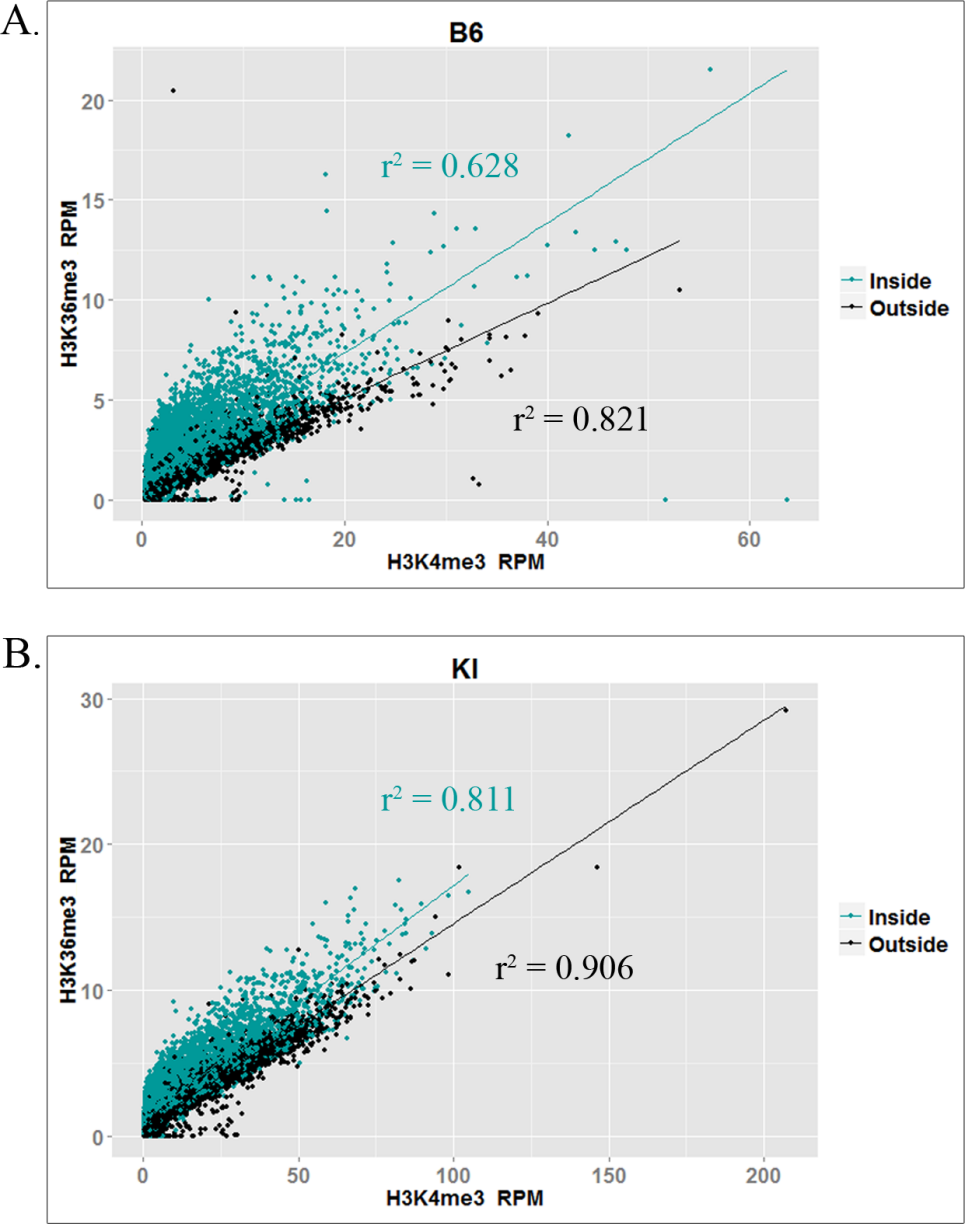
Correlation between H3K4me3 and H3K36me3 at hotspots. Plots show correlations between normalized (RPM) H3K4me3 and H3K36me3 ChIP-seq reads at individual hotspots in **(A)** the B6 strain and **(B)** the KI strain. Trendlines and correlation coefficients are shown for the subset of hotspots inside (teal) and outside (black) transcribed genes.

For each strain, we performed two biological replicates and merged the two datasets for analysis. Because H3K36me3 is enriched constitutively in actively transcribed genes by the histone methyltransferase SETD2 [26], we used the peak caller ZINBA [27] to call H3K36me3 peaks both inside and outside expressed genes (Table 1). In addition to calling both broad regions of enrichment (e.g. active genes) and narrow peaks (e.g. hotspots outside active genes), ZINBA can also call narrow peaks that show additional enrichment within broadly enriched regions (e.g. hotspots within active genes). Because PRDM9 trimethylates H3K36 at hotspots in active genes, which also contain SETD2-dependent H3K36 trimethylation, ZINBA enabled us to identify the additional PRDM9-dependent H3K36me3 enrichment at hotspots. We also compared the H3K36me3 data to H3K4me3 ChIP-seq data we previously reported for spermatocytes from the same strains [14]. Fig 2 shows the correlation between normalized H3K4me3 and H3K36me3 ChIP-seq reads in both strains at known B6 and KI hotspots, inside and outside transcribed genes. In both strains, the two marks are highly correlated at known hotspots, consistent with the idea that they are both placed there when PRDM9 binds at a hotspot. Correlations are higher at hotspots outside actively transcribed genes, which would be expected because hotspots inside active genes have varying levels of additional PRDM9-independent H3K36me3, which confounds the correlation. S4 Fig shows some representative examples of H3K4me3 and H3K36me3 in spermatocytes—at hotspots and elsewhere—and also shows examples of peaks called by ZINBA, at hotspots inside and outside actively transcribed genes.

**Table 1:** H3K36me3 ChIP-seq peaks at hotspots. This table presents the number of H3K36me3 peaks detected by ZINBA at hotspots, inside and outside actively transcribed genes. Peaks inside transcribed genes were detected as localized peaks above the broad, SETD2-dependent H3K36me3 enrichment seen in these genes.

	B6	KI
N Total Hotspots	18,848	28,474
N Hotspots Inside/Outside Transcribed Genes	8,377/10,471	11,316/17,158
N/% Hotspots with a detectable H3K36me3 Peak (ZINBA)	9,792/52.0%	17,373/61.9%
N Hotspots with a detectable H3K36me3 Peak (ZINBA) Inside/Outside Transcribed Genes	4,053/5,689	7,036/10,337
% Hotspots with a detectable H3K36me3 Peak (ZINBA) Inside/Outside Transcribed Genes	48.4%/54.3%	62.2%/60.2%

While the H3K36me3 ChIP-seq data show clear peaks at hotspots with a pattern similar to that of H3K4me3 (S4 Fig), enrichment is weaker for H3K36me3. Indeed, although the correlation between normalized H3K4me3 and H3K36me3 read counts at hotspots is very high (Fig 2), H3K4me3 enrichment is higher on average than H3K36me3 enrichment. As shown in Table 2, the degree of this difference in enrichment differs by strain, and between hotspots inside and outside transcribed genes in both strains, ranging between 1.81- and 3.74-fold, on average. This is consistent with our *in vitro* methylation results showing slower kinetics of H3K36 trimethylation by PRDM9 (Fig 1).

**Table 2:** H3K4me3/H3K36me3 ratio inside and outside actively transcribed genes. This table presents the ratio of normalized H3K4me3 ChIP-seq reads to normalized H3K36me3 ChIP-seq reads, inside and outside transcribed genes.

	B6	KI
N Total Hotspots	18,848	28,474
Mean/median H3K4me3/H3K36me3 ratio at hotspots inside transcribed genes (ZINBA)	1.81/1.53	2.80/2.24
Mean/median H3K4me3/H3K36me3 ratio at hotspots outside transcribed genes (ZINBA)	2.98/2.57	3.74/3.03

**Fig 3:**
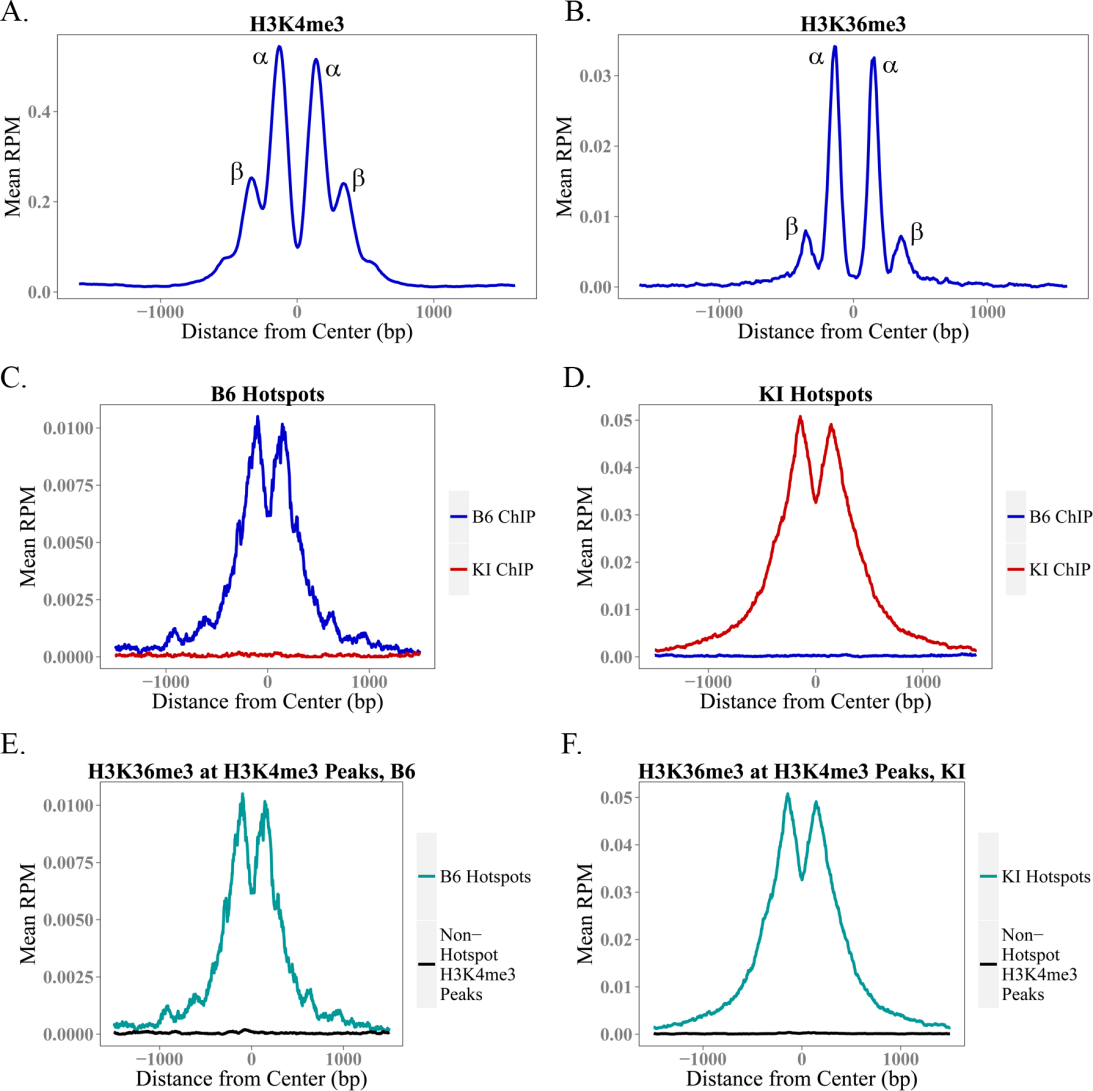
Aggregation Plots for H3K4me3 and H3K36me3 at Hotspots. **(A-B)** These aggregation plots show average H3K4me3 and H3K36me3 enrichment at 12,774 B6 hotspots for which we have previously determined the location of the 36bp PRDM9 binding site with high confidence. These plots, centered at the PRDM9 binding site, show the shape of the average H3K4me3 and H3K36me3 peak at a hotspot. **(C-D)** show average H3K36me3 enrichment across B6 (C) and KI (D) hotspots, using ChIP-seq data from the B6 and KI strains. Like PRDM9-dependent H3K4me3, H3K36me3 enrichment at hotspots is strain-specific. **(E-F)** show average H3K36me3 enrichment in hotspots specific to the denoted strain, and at non-hotspot H3K4me3 peaks (e.g. promoters). As measured by ChIP-seq, H3K4me3 and H3K36me3 coincide only at hotspots.

To compare the organization of the peaks for the two marks more precisely, we used previously determined data [28] on the location of the 36bp PRDM9 binding site at 12,774 hotspots in the B6 strain to generate aggregation plots of H3K4me3 and H3K36me3 ChIP-seq data centered on the midpoint of the PRDM9 binding site (Fig 3A and 3B). These plots show normalized ChIP-seq signal averaged across hotspots; we generated them using the Aggregation and Correlation Toolbox (ACT) software. [29] On average, H3K4me3 and H3K36me3 show similar spacing between the two α peaks (adjacent to the central nucleosome-depleted region on either side) and the two β peaks (adjacent to the alpha peaks) (Fig 3A and 3B), confirming that both are mediated by PRDM9 and suggesting that PRDM9 trimethylates H3K4 and H3K36 on the same nucleosomes. However, because the data are expressed as reads per million total reads and the totals include H3K4me3 and H3K36me3 peaks at sites other than hotspots, we cannot conclude from these data that PRDM9 trimethylates H3K4 more efficiently than H3K36, although the *in vitro* data reported above shows this to be the case for the PR/SET domain. What we can say is that because the ratio of methylation at α to β peaks differs for the two marks, PRDM9’s ability to trimethylate H3K36 relative to H3K4 decreases the further nucleosomes are from the central PRDM9 binding site.

To compare H3K36me3 at known H3K4me3 peaks at hotspots and elsewhere in the genome, we averaged the H3K36me3 signal within these two classes of H3K4me3 peaks, again using ACT [29]. Aggregation plots for H3K36me3 profiles at hotspots in both strains show that H3K36me3 peaks at hotspots are strain-specific (Fig 3C and 3D). Additionally, H3K36me3 is only observed at H3K4me3 peaks at hotspots; elsewhere in the spermatocyte genome, H3K4me3 and H3K36me3 are mutually exclusive (Fig 3E and 3F). Because we do not have the exact locations of the PRDM9 binding sites for all hotspots in both strains, and because PRDM9 does not bind at non-hotspot H3K4me3 peaks, for this analysis we centered the X-axis at the midpoint of each H3K4me3 peak and not at the center of the PRDM9 binding site. This blurs the shape of the peaks, but does not compromise their presence or absence.

### PRDM9-dependent H3K36me3 is independent of SETD2-dependent H3K36me3 at hotspots in actively transcribed genes

**Fig 4:**
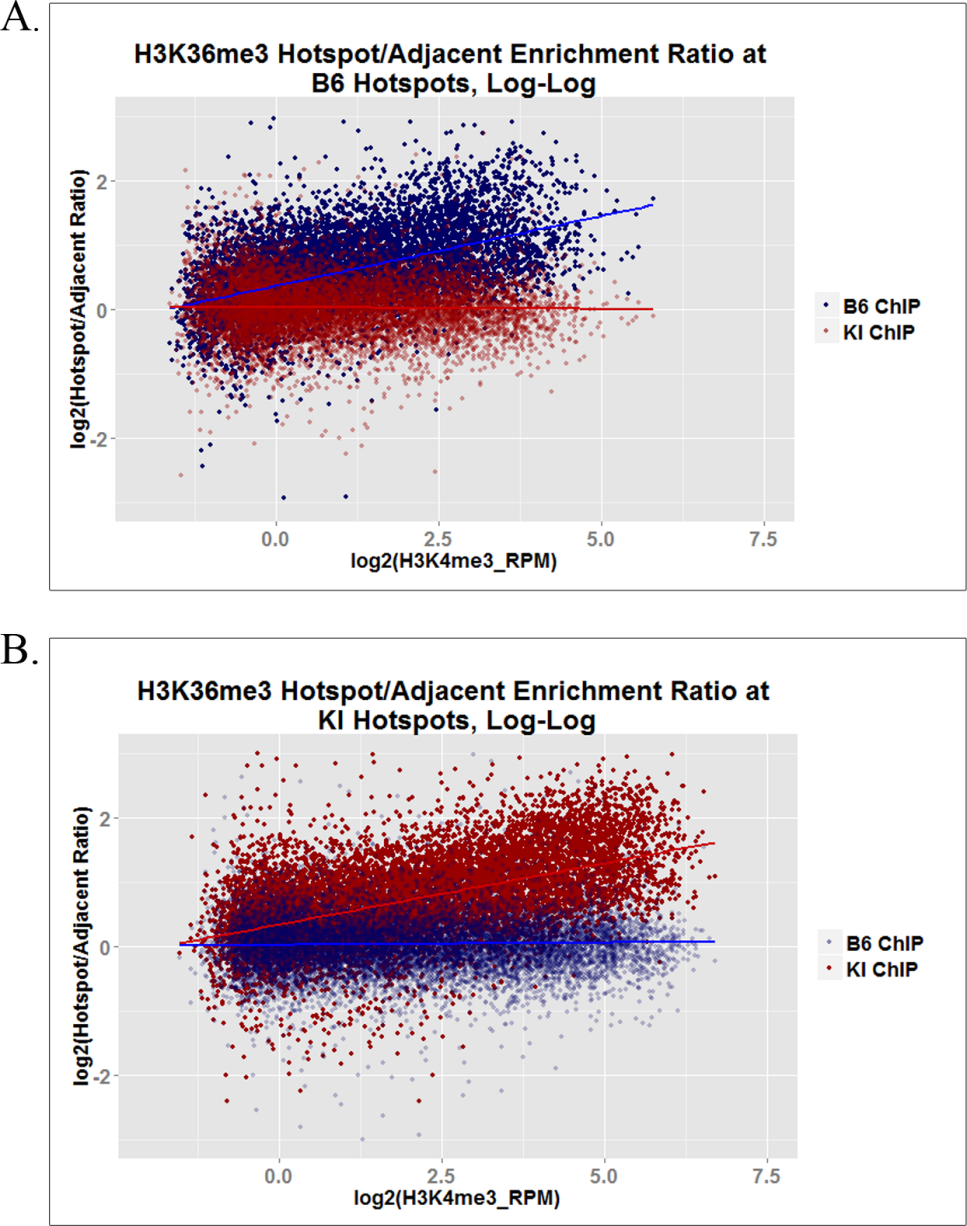
H3K36me3 inside and outside hotspots in actively transcribed genes. These log-log plots show, for hotspots located within actively transcribed genes, the ratio of normalized H3K36me3 ChIP-seq enrichment (RPM) within each hotspot to that within a same-sized region immediately adjacent to each hotspot, as a function of PRDM9-dependent H3K4me3 enrichment at each hotspot. **(A)** shows the data from both strains at B6 hotspots, while **(B)** shows the data from both strains at KI hotspots.

Using hotspots defined as H3K4me3 peaks not shared between B6 and KI, ZINBA detected an H3K36me3 peak at 48.4% and 62.2% of the hotspots in transcribed genes in B6 and KI, respectively (Table 1), suggesting that PRDM9 and SETD2 trimethylate H3K36 independently at these hotspots. We presume that the inability to detect H3K36me3 peaks at more of the hotspots in expressed genes reflects the difficulty in using ZINBA to recognize distinct H3K36me3 peaks within the elevated background of H3K36me3 in expressed genes. To confirm the existence and quantitate the enrichment of PRDM9-dependent H3K36me3 peaks in expressed genes, we compared H3K36me3 enrichment at hotspots with adjacent control regions of the same size in the same genes. We computed the ratio of H3K36me3 enrichment at each hotspot to that of its corresponding adjacent control region, and plotted the results as a function of H3K4me3 enrichment. This plot (Fig 4) shows that an additional, strain-specific H3K36me3 signal is present at some hotspots in actively transcribed genes, and that, not surprisingly, our ability to detect hotspots with an additional H3K36me3 signal increases with PRDM9-dependent H3K4 trimethylation.

Because H3K36me3 ChIP-seq describes the collective properties of several million cells and the same histone molecule cannot be trimethylated at K36 by both PRDM9 and SETD2, this additional enrichment at hotspots means that each enzyme is trimethylating only a fraction of histone molecules present at those sites, allowing the two reactions to appear additive. S4 Fig shows some examples of hotspots in an actively transcribed gene, which have an additional PRDM9-dependent peak, as viewed in the UCSC Genome Browser.

### PRDM9 trimethylates H3K4 and H3K36 on the same histone molecules *in vitro* and the same nucleosomes *in vivo*, and is almost solely responsible for the H3K4me3/H3K36me3-double-positive nucleosomes found in spermatocytes

**Fig 5:**
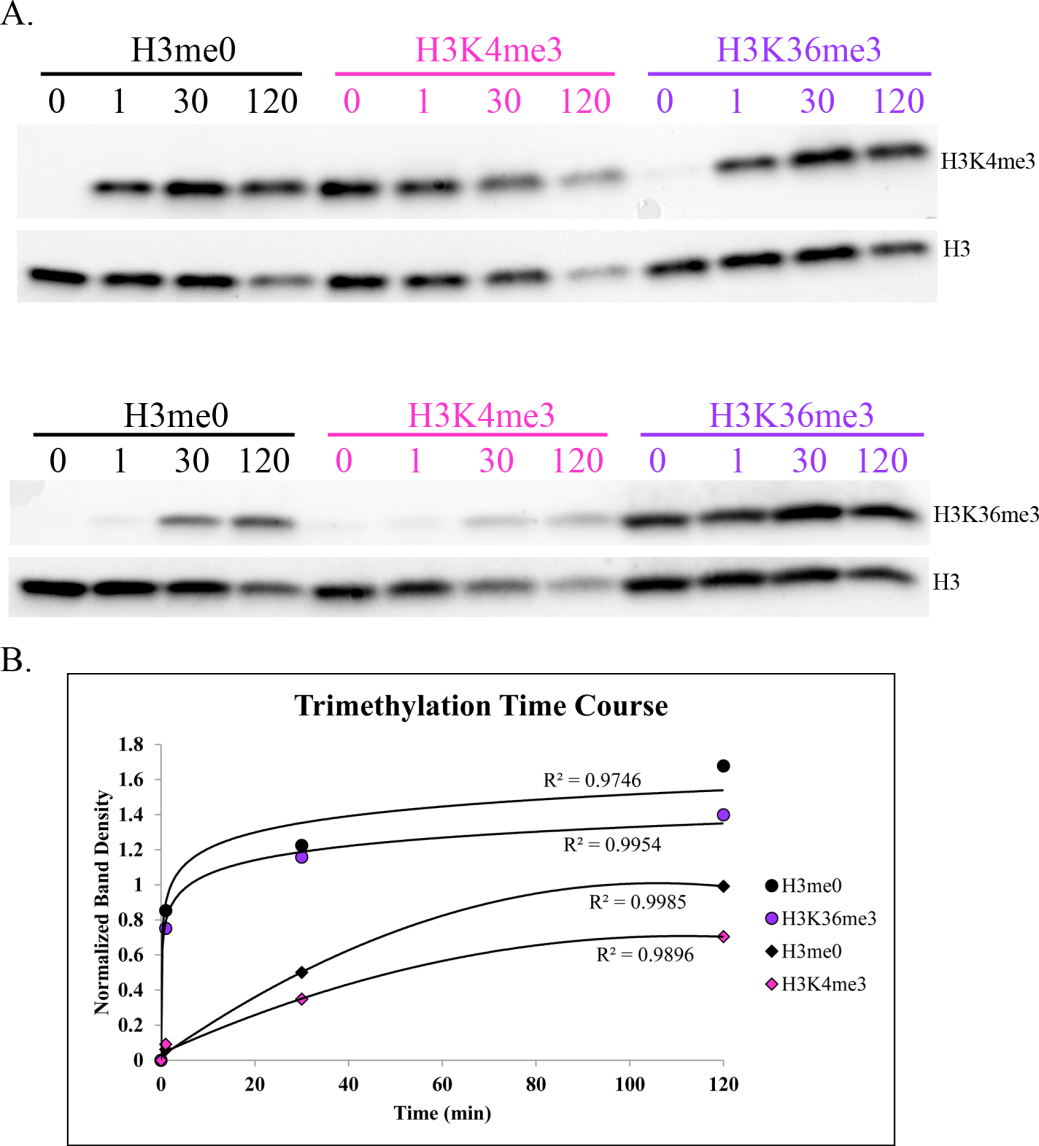
Intrinsic ability of PRDM9 PR/SET domain to trimethylate H3K4 and H3K36 on histones previously trimethylated for the other mark. **(A)** Western blots of a single histone methyltransferase assay time course, measuring the H3K4 and H3K36 trimethylation activity of recombinant PRDM9 PR/SET domain, using recombinant unmodified histone H3 (black) H3K4me3 (pink) or H3K36me3 (purple) as substrate. Duplicate blots were probed with α-H3K4me3 and α-H3K36me3 antibodies, respectively. Both blots were then stripped and re-probed with α-Histone H3 antibody. Numbers indicate time in minutes. **(B)** Methyltransfease assay time course, quantified by densitometry and normalized to histone H3 as in Fig 1. Circles represent H3K4 trimethylation, while diamonds represent H3K36 trimethylation, on the histones denoted in the legend.

To determine whether the PRDM9 PR/SET domain is capable of trimethylating both K4 and K36 on the same histone H3 molecule, we tested the trimethylation activity of its PR/SET domain *in vitro* separately with H3K4- and H3K36-trimethylated histones as substrates. We found that the PRDM9 PR/SET domain is capable of trimethylating H3K4 on previously H3K36-trimethylated histones, and H3K36 on previously H3K4-trimethylated histones (Fig 5). The ChIP-seq data suggest that PRDM9 trimethylates H3K4me3 and H3K36me3 on the same nucleosomes *in vivo* (Fig 3A and 3B).

**Fig 6:**
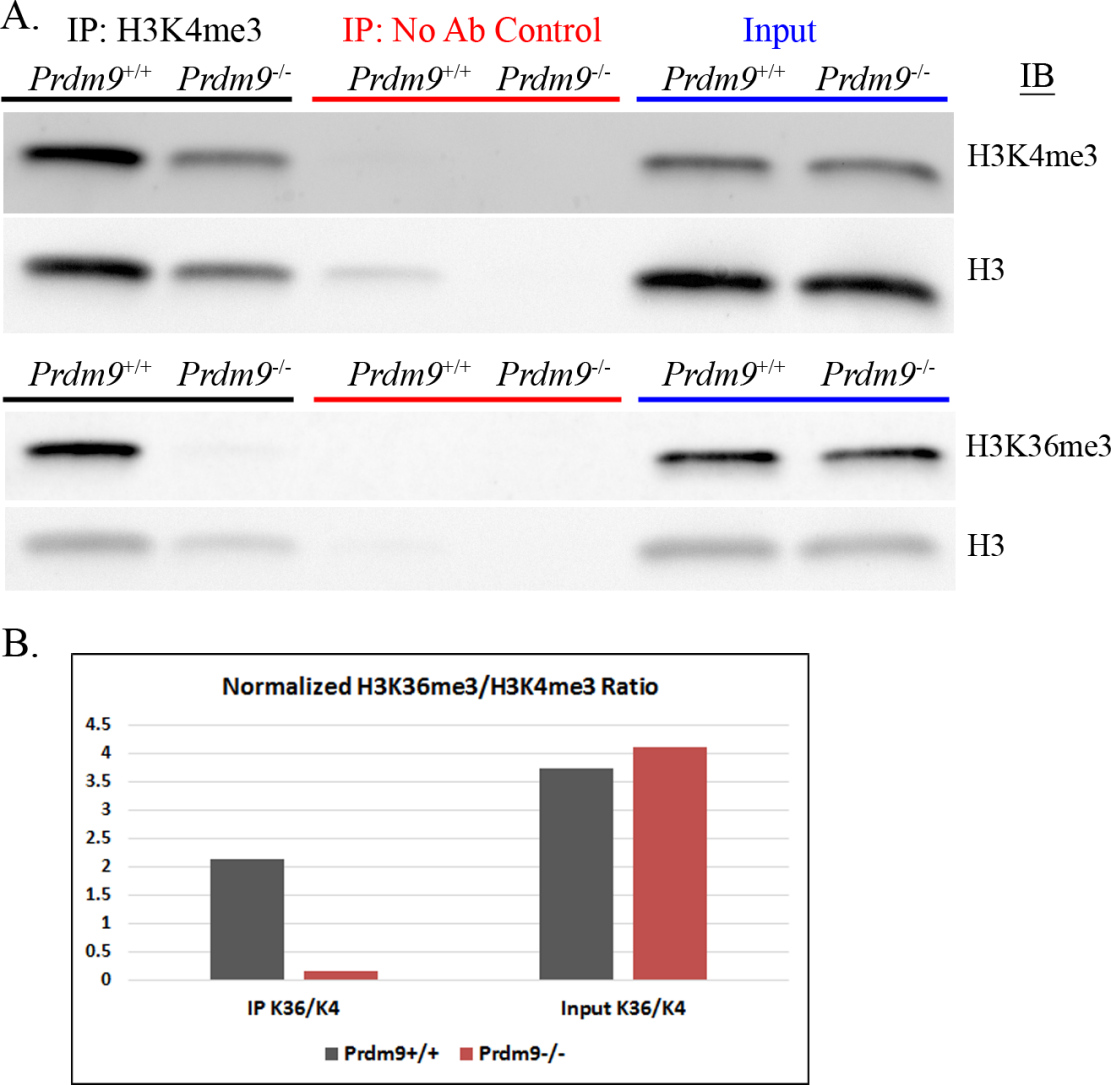
Detection of H3K4me3/H3K36me3-double-positive nucleosomes *in vivo* by immunoprecipitation. **(A)** This figure shows immunoprecipitation and immunoblotting of histones extracted from 14dpp mouse spermatocytes, from either C57BL/6J mice *(Prdm9*^+/+^) or homozygous B6;129P2-*Prdm9*^tm1Ymat^/J mice *(Prdm9*^-/-^). Immunoprecipitation was done using 4μg acid-extracted histone, with either α-H3K4me3 antibody or no antibody as a control. Input samples were prepared with 0.5μg acid-extracted histone. Equal amounts of each IP and Input were run on two identical gels, which were transferred to nitrocellulose and immunoblotted with either α-H3K4me3 or α-H3K36me3 antibody. Both blots were then stripped and re-probed with α-Histone H3 antibody to confirm equal loading of total histone. See S5 Fig for a biological replicate with an additional re-probe for histone H4. **(B)** This chart shows the ratios of H3K36me3 signal to H3K4me3 signal, as quantified by densitometry, in the *Prdm9*^+/+^ and *Prdm9*^-/-^ IPs and inputs. The H3K4me3 and H3K36me3 bands for both the IPs and inputs were normalized to the histone H3 band in the corresponding input.

To confirm the presence of H3K4me3/H3K36me3-double-positive nucleosomes in spermatocytes, and to determine whether they are dependent on PRDM9, we purified histones by acid-extraction from germ cells isolated from 14dpp wild-type B6 animals, and from *Prdm9*-null animals (homozygous B6;129P2-*Prdm9*^tm1Ymat^/J [19]). We then immunoprecipitated histones with α-H3K4me3 antibody, and immunoblotted with α-H3K4me3 and α-H3K36me3 antibodies (Fig 6, S5 Fig). We found that H3K4me3/H3K36me3-double-positive nucleosomes are present in germ cells with functional PRDM9, but dramatically reduced when PRDM9 is absent, confirming that this double-positive signature is both unique to hotspots and almost entirely dependent on PRDM9. Despite the fact that equal amounts of total histone were added to the IPs, the amount of H3K4 trimethylated histone that immunoprecipitated from *Prdm9*^-/-^ spermatocytes is markedly less than that from wild-type B6 spermatocytes. This is expected; during the leptotene-zygotene phases of prophase I, PRDM9-dependent H3K4me3 at hotspots accounts for a substantial proportion of total H3K4me3 in the nucleus. When this is removed in *Prdm9*^-/-^ cells, a congruently smaller proportion of the histones in those cells will be H3K4 trimethylated, and less is available for immunoprecipitation. This difference is probably not obvious in the input lanes due to the semi-quantitative nature of Western blotting when smaller amounts of protein are present. The H3K4me3 enrichment in the IP lanes has likely amplified the difference in total H3K4me3 between the two genotypes and made it more visible on a Western blot. Likewise, the absence of PRDM9-dependent H3K4me3 in the *Prdm9*^-/-^ histone preparation entails a lower concentration of H3K4me3 relative to wild-type, which may have reduced the efficiency of the *Prdm9*^-/-^ IP. In any case, Fig 6B shows that the ratio of H3K36me3 signal to H3K4me3 signal on immunoprecipitated histones is reduced 13-fold in *Prdm9*^-/-^ spermatocytes relative to B6 spermatocytes, demonstrating that the vast majority of H3K4-trimethylated histones are not H3K36-trimethylated in the absence of PRDM9.

Although the ChIP data show that HK4me3 and H3K36me3 do not coincide outside hotspots in spermatocytes (Fig 3E and 3F), there is still a small amount of double-positive signal in the *Prdm9*^-/-^ IP (Fig 6, S5 Fig). This is likely explained by the pseudoautosomal region (PAR) on the X and Y chromosomes, where there is an obligate crossover in male meiosis that has been shown to be PRDM9-independent [13]. Most of the PAR contains highly repetitive sequence, resulting in very low or no mappability for ChIP-seq data; however, there is one non-repetitive segment at the beginning of the PAR where it was possible to map reads (S6 Fig). Interestingly, we observed an abrupt and substantial increase in both H3K4me3 and H3K36me3 in this region, with a concomitant, sharp increase in meiotic DSB activity as measured by the DMC1 ChIP-seq data from Brick et al. (2012) [13]. The abundant PRDM9-independent double-positive chromatin in this region would account for the small amount of double-positive nucleosomes in the *Prdm9*^-/-^ IP, but not in the ChIP data, as most of the region is unmappable. The fact that this PRDM9-independent site of recombination is also double-positive for H3K4me3 and H3K36me3 provides further evidence in favor of the idea that this chromatin signature is required for successful meiotic DSB targeting and/or repair.

S5 Fig shows a biological replicate of this experiment; additionally, this blot was stripped and re-probed for histone H4. The presence of a very faint histone H4 band (arrow) shows that at least some of the K4-trimethylated histone H3 in the *Prdm9*^+/+^ IP co-immunoprecipitated with histone H4. This band is likely undetectable in the *Prdm9*^-/-^ IP because of the lower amount of total histone that was immunoprecipitated. Although the ChIP data show that PRDM9 trimethylates H3K4 and H3K36 on the same nucleosomes (Fig 3A and 3B), and the PRDM9 PR/SET domain is able to trimethylate both residues on the same histone *in vitro* (Fig 5), the presence of histone H4 in the *Prdm9*^+/+^ IP shows that some dimers or multimers are present in the acid-extracted histones. Because the amount of detectable histone H4 in the IP is almost negligible, it is likely that PRDM9 trimethylates K4 and K36 on at least some of the same histone molecules *in vivo*, but we cannot conclude this definitively and have made the conservative conclusion that PRDM9 trimethylates H3K4 and H3K36 on the same nucleosomes at hotspots.

Together, these data provide strong evidence that PRDM9 trimethylates H3K4 and H3K36 on the same nucleosome, and suggest that it likely does so on the same histone molecule *in vivo*.

### The H3K36me3/H3K4me3 ratio at nucleosomes is independent of H3K4me3 enrichment level

**Fig 7:**
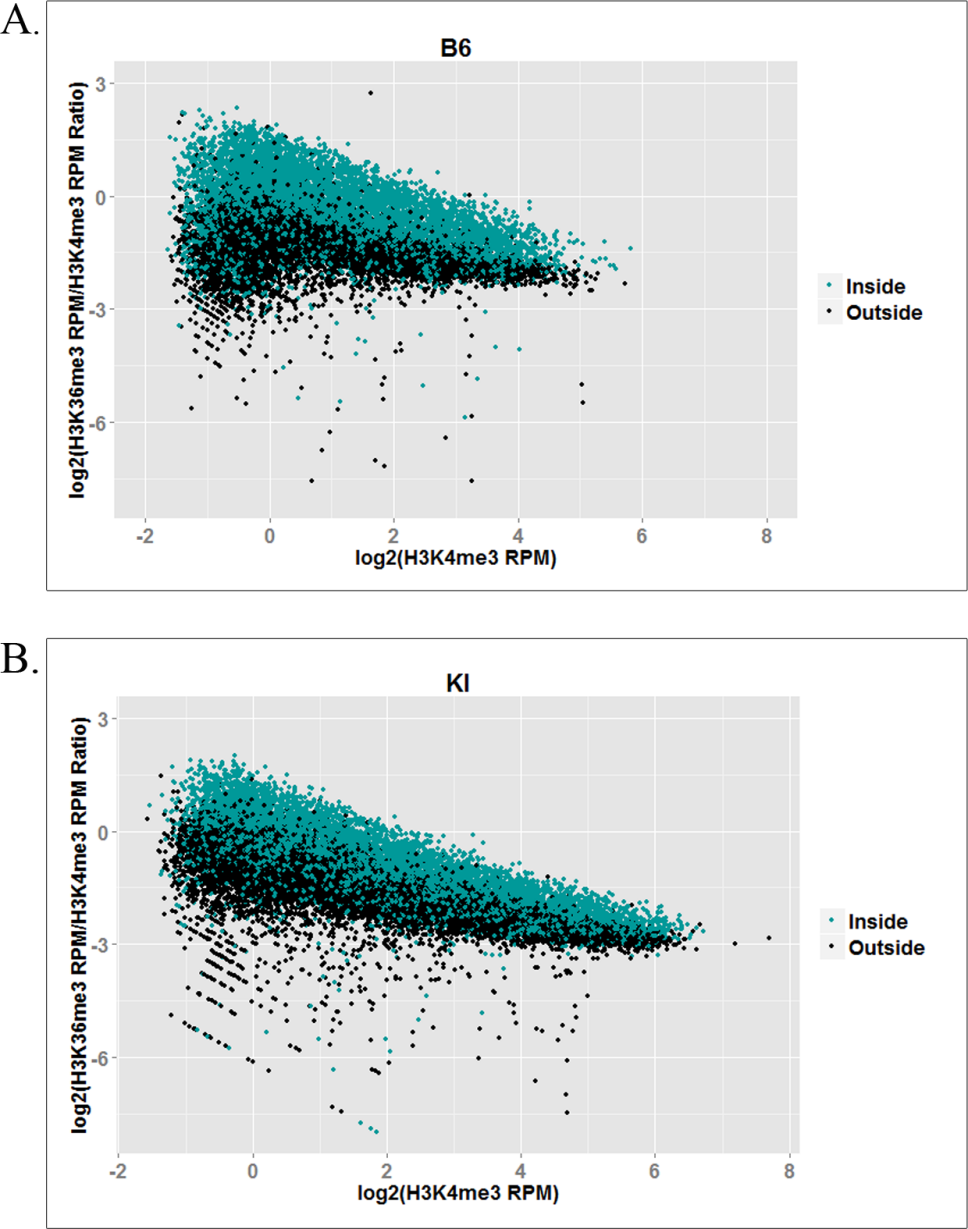
Log-log plots showing the ratio of H3K36me3 to H3K4me3 at hotspots as a function of PRDM9-dependent H3K4me3 enrichment, inside and outside actively transcribed genes. **(A)** shows the B6 hotspot data in the B6 strain; **(B)** shows the KI hotspot data in the KI strain.

H3K4me3 enrichment at hotspots describes the relative frequency with which each hotspot is trimethylated by PRDM9 *in vivo* [14], and is correlated with its probability of acquiring a DSB. [13] Importantly, whatever the mechanisms controlling these frequencies may be, they have little influence on the H3K36me3/H3K4me3 ratio (Fig 7), indicating that once bound at a hotspot the ability of PRDM9 to trimethylate H3K4 and H3K36 is relatively constant. While we do observe a modest decrease in this ratio with increasing H3K4me3, this is likely due to the intrinsically higher background level of H3K36me3 ChIP-seq data compared to H3K4me3 ChIP-seq data, especially inside actively transcribed genes (S4 Fig). As PRDM9-dependent H3K4me3 increases, PRDM9-dependent H3K36me3 also increases (Fig 2), rendering the background level of H3K36me3 progressively less predominant in the ratio. This implies that the H3K36me3/H3K4me3 ratio at higher-trimethylation hotspots approximates PRDM9’s intrinsic H3K4 and H3K36 trimethylation activities more closely than that at lower-trimethylation hotspots. This would also account for the somewhat steeper decrease in the H3K36me3/H3K4me3 ratio observed in the KI strain (Fig 7B) compared to the B6 strain (Fig 7A). Since KI hotspots show higher trimethylation overall than B6 hotspots (Fig 2), they would be expected to transcend the H3K36me3 background to a more pronounced degree.

## Discussion

The data presented here show that PRDM9 places two epigenetic marks on the same nucleosomes at hotspots *in vivo*, H3K4me3 and H3K36me3, and that the H3K4me3/H3K36me3-double-positive signature is exclusive to hotspots and driven almost entirely by PRDM9 in spermatocytes, with the PRDM9-independent PAR region being the only exception we detected. Our *in vitro* data also establish that full-length PRDM9 recapitulates and extends previous observations on the methyltransferase activities of the isolated PR/SET domain. The kinetic data from *in vitro* assays show that with free histone H3 as substrate the PR/SET domain of PRDM9 trimethylates H3K4 much more rapidly than H3K36 (Fig 1), which is consistent with previously published data on the human PRDM9 PR/SET domain with histone H3 peptides as substrate [20]. The peptide array data also show that full-length PRDM9 can methylate H3K9, as was previously reported for the PR/SET domain [21]. For all three residues—K4, K9 and K36—their monomethylated state is the poorest substrate for PRDM9 (S3A Fig).

Whether PRDM9-dependent methylation of H3K9 also occurs *in vivo* is an open question as there is now evidence that acetylated K9 is associated with recombination hotspots in yeast [30], and we have now shown that acetylation at H3K9 prevents subsequent methylation (S3C Fig). The possible role of phosphorylation of H3T3 and H3T11 in meiosis is not known. Phosphorylation of H3S10 is a known marker of the meiotic G2/MI transition [31], but its presence during early leptotene-zygotene when PRDM9 is most active is uncertain.

The ChIP-seq data from spermatocytes show, on average, a 1.81- to 3.74-fold excess of H3K4me3 over H3K36me3 at hotspots (Table 1), but the true ratio cannot be established using ChIP-seq data alone as hotspots do not represent the same fraction of all trimethylation for H3K4me3 and H3K36me3. In addition to hotspots, H3K36me3 is enriched throughout the gene bodies of all actively transcribed genes [32], and additional H3K36me3 is deposited in the G1 and early S phases of the cell cycle [33]. H3K4me3 outside hotspots, on the other hand, is enriched at active and poised promoters and enhancers [34]. Consequently, the ChIP-seq enrichment patterns and signal-to-noise ratios are intrinsically very different for H3K4me3 and H3K36me3 (see S4 Fig for a visual representation). However, the difference in enrichment between the α and β peaks of the average hotspot does suggest that this difference in activity is likely to exist *in vivo* as well. The β/α ratio is much lower for H3K36me3 (Fig 3A and 3B), suggesting that in the time PRDM9 remains bound at a hotspot it is much less able to trimethylate H3K36 than H3K4 on the β nucleosomes, or that the H3K36me3 mark is simply more transient than the H3K4me3 mark, and persists for a shorter period of time.

There are also some differences in H3K36me3 deposition at hotspots between the B6 and KI strains. The aggregation plots in Fig 3C and 3D show that there is higher H3K36me3 enrichment at the average hotspot in KI, relative to B6. Fig 2 shows that enrichment for both H3K4me3 and H3K36me3 is higher at KI hotspots than at B6 hotspots, corroborating previously published data in these two strains for H3K4me3 [35], and the correlations between the two marks are slightly stronger, especially in actively transcribed genes. These results corroborate the model of Baker et al. regarding hotspot erosion, the process by which hotspots gradually lose their activity in mammals [35]. Under this model, gene conversion reduces the affinity of an allele of PRDM9 for its complement of hotspots over evolutionary time. This, in turn, reduces the activity of the hotspots as measured by H3K4me3, because PRDM9 now either binds at eroded hotspots less often, or remains bound for a shorter period of time, or both. Eventually, this loss of activity drives positive selection for new alleles of *Prdm9*, which reset the cycle [35]. The B6 and KI strains are genetically identical except for the zinc finger array of PRDM9. This genetic background has been exposed to the endogenous *Prdm9*^Dom2^ allele for many thousands of generations, allowing this allele’s hotspots to erode. By contrast, when the *Prdm9*^Cst^ allele was knocked in to the B6 background, it was effectively a new allele. The non-eroded hotspots in the KI strain show higher enrichment for both H3K4me3 and H3K36me3, as would be expected given the strong correlation between the two marks and the fact that both are PRDM9-dependent at hotspots.

The PR/SET domain of PRDM9 is remarkably self-sufficient and promiscuous in its catalytic functions, features that are not typical of a metazoan histone methyltransferase, let alone a mammalian one. The ability of PRDM9 to carry out so many different enzymatic reactions by itself using the same active site (sequential mono-, di-, and trimethylation of H3K4 and H3K36 and possibly of H3K9 *in vivo*) is unusual for a mammalian histone methyltransferase, where “division of labor” (e.g. each enzyme or complex performs one or two reactions) appears to be the general rule [23,36]. SETD2, for instance, is responsible for all or nearly all of the H3K36me3 associated with transcription, yet it appears to catalyze only methylation of H3K36me2 *in vivo*, unlike its yeast homologue SET2, which performs all three methylation reactions sequentially. [26,36,37]. In mammals, this transcription-associated H3K36 trimethylation is multi-step, involving multiple proteins, and thus available for fine-tuned regulation at each step [36]. This does not appear to be true of PRDM9-dependent H3K4me3 and H3K36me3, whose tight correlation at hotspots (Fig 2) indicates that any regulation these histone marks are subject to broadly affects both marks at once.

PRDM9’s associated histone marks are essential for mammalian meiosis to succeed [13,19], and it is thus not surprising that the structure of its PR/SET domain is highly conserved in mammalian evolution. By contrast, the zinc finger domain of *Prdm9*, which is responsible for determining hotspot locations, may be the most rapidly evolving mammalian protein-coding sequence known; with well over a hundred alleles described in wild mouse populations [38,39], and at least 30 in humans [40,41]. Having distinct exons encode these two diverse functions—trimethylation and DNA binding—allows the zinc finger domain to evolve more freely and more rapidly than a tighter genetic linkage might otherwise permit. Indeed, this rapid evolution of the zinc finger domain is an essential feature of PRDM9 function; it generates new *Prdm9* alleles whose protein products bind new complements of hotspots, thereby overcoming the consequences of hotspot erosion and enhancing genetic diversity. However, this does leave open the question of the roles that PRDM9’s multiple catalytic functions play in meiosis.

PRDM9 defines recombination hotspots in most mammals, with the exception only of canids to date. However, PRDM9 clearly does more than select the genomic loci where recombination will occur; several lines of evidence indicate that PRDM9 directly or indirectly influences the repair of meiotic double-strand breaks. Once DSBs have formed in normal meiosis, the span of Holliday junction branch migration during DSB repair is limited to the span of nucleosomes marked by PRDM9-driven H3K4me3 [14], and the mis-localized DSBs present in the *Prdm9* null mutant fail to be repaired properly [13,19]. Finally, the ectopic DSBs that do occur in the *Prdm9* null mutant frequently localize at non-hotspot sites of H3K4me3, such as promoters [13], indicating that some signal in addition to H3K4me3 is required to direct the correct localization and repair of DSBs.

Logically, the most obvious possibilities for what these directing marks might be are the combination of H3K4me3 and PRDM9, the combination of H3K4me3 and H3K36me3, or the combination of all three. Several clues suggest that the combination of H3K4me3 and H3K36me3 is the most likely possibility. One is that although H3K4me3 and H3K36me3 occur abundantly elsewhere in germ cells, they coincide only at hotspots and within the PAR. Another is that DSBs occur most frequently close to or within the PRDM9 binding site, as evidenced by the accumulation of mutations due to gene conversion at historically active hotspots [14,28]. This indicates that DSB formation likely occurs after PRDM9 exits hotspots. The fact that Holliday Junction migration correlates with the span of H3K4-trimethylated nucleosomes [14] also indicates that these marks remain after PRDM9 has exited. Finally, H3K4me3/H3K36me3-double-positive chromatin is present in the PAR, a region of PRDM9-indepenedent recombination, uniting all regions of mammalian recombination under a distinct chromatin signature. These lines of evidence suggest that joint trimethylation at K4 and K36 is required for targeting DSBs to hotspots. Additionally, recent evidence from cell culture systems that H3K36me3 is required for DSB repair in somatic cells by HRR [24,25], together with the fact that meiotic DSBs are repaired exclusively by HRR [18,42], suggest that H3K36me3 may also play a role in recruiting the DSB machinery. Further work is obviously necessary to determine the exact role of PRDM9-dependent H3K36me3 at hotspots; if it does have a role in meiotic DSB targeting and/or repair, it will explain the meiotic arrest in *Prdm9*-null mice, why PRDM9 is the only known mammalian histone methyltransferase capable of *de novo* trimethylation of both H3K4 and H3K36, and why PRDM9 places both of these otherwise mutually exclusive marks simultaneously at hotspots.

## Materials and Methods

### Ethics statement

The animal care rules used by The Jackson Laboratory are compatible with the regulations and standards of the U.S. Department of Agriculture and the National Institutes of Health. The protocols used in this study were approved by the Animal Care and Use Committee of The Jackson Laboratory (Summary #04008). Euthanasia for this study was done by cervical dislocation.

### Mouse strains

Strains used are the following: C57BL/6J (stock number 000664), B6.Cg-*Prdm9*^<tm1.1Kpgn^>/Kpgn, and B6;129P2-*Prdm9*^tm1Ymat^/J. The B6.Cg-*Prdm9*^<tm1.1Kpgn^>/Kpgn (formerly designated as B6-Prdm9^CAST-KI^) and B6;129P2-*Prdm9*^tm1Ymat^/J strains were generated and characterized as described previously. [14,19]

### H3K36me3 ChIP-seq

H3K36me3 ChIP was performed using the same protocol we reported previously for H3K4me3 ChIP [14], using a commercially available polyclonal α-H3K36me3 (Active Motif, cat#61101; https://www.activemotif.com/). DNA samples were sequenced on an Illumina HiSeq 2500, with 100bp reads, and trimmed for quality using trimmomatic. Sequence data were aligned to the mouse mm9 genome using BWA v1.2.3, and reads which failed to align to unique positions in the genome were discarded. Two biological replicates were done for each strain, resulting in 97,152,930 and 54,392,016 aligned reads for B6, and 108,384,029 and 91,262,924 for KI. One input sample for each strain was also sequenced, resulting in 72,524,718 aligned reads for B6 and 77,681,045 aligned reads for KI. After alignment and filtering, the two ChIP replicates for each strain were combined for analysis. Data are available at NCBI Gene Expression Omnibus (GEO; http://www.ncbi.nlm.nih.gov/geo) under accession number GSE76416. Hotspot locations for both the B6 and KI strains were previously described by Baker et al. [14]; files with these hotspot locations are available under GEO accession number GSE52628.

### H3K36me3 peak calling

H3K36me3 broad (unrefined) and narrow (refined) peaks were called using ZINBA v2.0.2.03. Parameters were: Winsize = 1000, Offset = 500, pWinSize = 750, WinGap = 250, Type = complete, and FDR = False. All other parameters were default. The input samples were used as controls. ZINBA refined peaks overlapping known hotspots were ascertained using bedtools (v2.22.0) intersect. Genes were designated actively transcribed or inactive based on previously reported RNA-seq data from germ cells extracted from testes of B6 mice at 12dpp. [28] The RNA-seq data are available under GEO accession number GSE61613.

### H3K4me3 ChIP-seq data and peak calling

ChIP-seq data for both the B6 and KI strains were previously reported by Baker et al. (2014) [14] for H3K4me3 peaks. Data are available under GEO accession number GSE52628. The H3K4me3 peak set reported by Baker et al. (2014) [14] was also used for this study.

### Aggregation plots

Analyses for the aggregation plots were carried out using the Aggregation and Correlation Toolbox (ACT, http://act.gersteinlab.org/) [29]. ACT parameters were: nbins = 500, mbins = 0, radius = 1500. H3K4me3 peaks inside and outside known hotspots were determined using bedtools (v2.22.0) intersect.

### Western blotting

Transfer to nitrocellulose was conducted either overnight at 22V in transfer buffer (1X tris-glycine with 20% methanol) by standard wet blotting procedures (Figs 1 and 5), or with an iBlot dry blot transfer apparatus (ThermoFisher, https://www.thermofisher.com/) according to the manufacturer’s protocol (Fig 6).

For immunoblotting, blots in Figs 1 and 5 were blocked at for 1 hour in 15mL blocking buffer—10mg/mL bovine serum albumin (BSA) in tris buffered saline with 0.3% tween (TBST). They were then incubated with 15mL primary antibody solution for 1 hour. Blots were then rinsed with TBST and given 3 5-minute washes in TBST. 15mL secondary antibody was then added, and the blots were incubated for 1 hour. They were then rinsed with TBST and given 2 15-minute washes in TBST. After the final wash, 4mL of Femto West ECL (ThermoFisher, https://www.thermofisher.com/) were added to each blot; the blots were incubated 1 minute in the ECL, then imaged with a G:BOX documentation system (SynGene, http://www.syngene.com/). Densitometry was performed using GeneTools software (SynGene, http://www.syngene.com/).

The blot in Fig 6 was immunoblotted using a SNAP id 2.0 protein detection system (EMD Millipore, http://www.emdmillipore.com/), according to the manufacturer’s instructions. After the final wash, the blot was incubated in Femto West ECL and imaged by the same procedure as the blots in Figs 1 and 5.

After probing for H3K4me3 or H3K36me3, all blots were stripped and re-probed with an α-Histone H3 antibody. Stripping was carried out for 10 minutes in Restore Western Blot Stripping Buffer (ThermoFisher cat#21059, https://www.thermofisher.com/), after which the blots were rinsed in TBST and then given two 15-minute washes in TBST, then blocked for 1 hour in blocking buffer.

All incubations were done at room temperature, and with the exception of the ECL incubations, with gentle shaking on a rotating platform. All antibody solutions consisted of antibody diluted in blocking buffer at the indicated concentrations.

### Peptide array

#### Methylation analysis on histone peptide arrays

Full-length mouse PRDM9 (mPRDM9) was expressed in E. coli fused to an N-terminal MBP tag. The fused protein (130 kDa) was subjected to two-step purification on SP-sepharose and amylose beads. The final protein prep was concentrated to 0.5 mg/ml and contained >85% MBP-mPRDM9 determined by densitometry analysis of silver-stained SDS gels (S1 Fig). The purified protein showed H3K4 methyltransferase activity as measured by western blotting (S1 Fig).

Histone peptide arrays (cat#13005) were purchased from Active Motif, Carlsbad, CA (www.activemotif.com). The arrays were pre-incubated with 1X methylation buffer (50mM Tris pH9, 100mM NaCl, 10μM ZnCl2, 0.05% beta-mercaptoethanol) for 20 min. The preincubation buffer was removed and the arrays were incubated with 1X methylation buffer with added mPRDM9 (42 ng/μl or 125 ng/μl) and 1 μM (^3^H-methyl)S-adenosylmethionine, for 30, 60 or 90 min. The arrays were washed four times with 50mM NH4HCO3, 0.1% SDS, and then washed four times with PBS for 5 min each time.

The arrays were dried completely and exposed for 4 days, then the images were scanned and analyzed using the Array Analysis Software provided by the manufacturer (https://www.activemotif.com/catalog/668/modified-histone-peptide-array).

### Expression and purification of PRDM9 PR/SET domain

A cDNA encoding amino acids 192–377 of murine PRDM9 was synthesized and cloned into the pBAD/His B expression vector (ThermoFisher, https://www.thermofisher.com/) by GenScript’s gene synthesis service (http://www.genscript.com/). This vector was transformed into One Shot TOP10 chemically competent *E*. *coli* (ThermoFisher, https://www.thermofisher.com/), following the manufacturer’s protocol. The transformed vector was purified from a 10mL culture using a QIAprep Spin Miniprep kit (Qiagen, https://www.qiagen.com/), and the insert Sanger-sequenced (GeneWiz, http://www.genewiz.com/) to ensure there were no mutations. A 500mL culture was grown in LB broth with 50μg/mL carbenicillin at 37°C for ~5 hours, to an optical density of ~0.5; expression of the PR/SET domain was then induced by adding arabinose to a concentration of 0.02%, and incubating the culture at 14°C for 24 hours. Cells were then pelleted and stored at -80°C for at least 24 hours. The pellet was thawed and resuspended in 10mL 1X Native Purification Buffer (50mM NaH_2_PO_4_, 0.5M NaCl, pH = 8.0), then lysozyme (Sigma-Aldrich, https://www.sigmaaldrich.com/) was added to 1mg/mL and the suspension incubated on ice for 30 minutes. Phenylmethanesulfonyl fluoride (PMSF) was then added to 1mM, and protease inhibitor cocktail (PIC) (Sigma, https://www.sigmaaldrich.com/) was added to 1X. The cell suspension was then sonicated on ice, using a MiSonix 3000 sonicator (Cole-Parmer, http://www.coleparmer.com/), with 9 30-second pulses with 3-minute intervals between each pulse, at an amplitude of 6. After sonication, the suspension was centrifuged for 30 minutes at 10,000xg and 4°C to remove insoluble debris. The supernatant was then mixed with 0.5mL ProBond nickel-chelating resin (ThermoFisher, https://www.thermofisher.com/), which was equilibrated according to the manufacturer’s protocol. This mixture was incubated for 1 hour at room temperature with gentle rotation to bind the 6xHis-tagged recombinant protein to the resin. It was then transferred to a column, and the resin settled by a brief low-speed centrifugation (800xg). The column was clamped, and the supernatant was allowed to flow through at 4°C. The column was then washed 4 times, each with 8mL 1X Native Purification Buffer (4°C). After the final wash, the resin was eluted with 4mL Native Elution Buffer (Native Purification Buffer with 250mM imidazole, pH = 8.0), in 8 0.5mL fractions. The fractions were stored at 4°C until the following day. The next day, the presence of recombinant PRDM9 PR/SET domain in the fractions was checked by Western blotting, using the Xpress antibody (ThermoFisher cat#R910-25, https://www.thermofisher.com/) against a tag incorporated by the pBAD/His B vector, at a 1:3000 dilution. Most of the recombinant protein had been eluted in the first three fractions; these were combined and dialyzed overnight against 1L protein storage buffer (20mM K_2_HPO_4_, 0.5mM EDTA, 75mM KCl, 10% glycerol, pH = 7.5), then for an additional 4 hours against a fresh liter of protein storage buffer on the following day, at 4°C. PMSF and PIC were added to the resulting partially purified recombinant PR/SET domain, to concentrations of 1mM and 1X, respectively. Total protein was quantified via Bradford assay, and the solution was aliquoted and stored at -80°C.

### Histone methyltransferase assays

Recombinant histones were supplied by Active Motif (https://www.activemotif.com/): unmodified histone H3 (cat#31207), H3K4me3 (cat#31210), and H3K36me3 (cat#31219). All histones were dissolved in water to a concentration of 1μg/μl, aliquoted, and stored at -80°C. Histone methyltransferase assays were carried out at 23°C in a water bath, and consisted of 33.4ng/μl recombinant PRDM9 PR/SET domain, 10ng/μl recombinant histone, 5μg/μl S-adenosylmethionine (SAM), in 1X HMT buffer (25mM Tris, 10% glycerol, 1mM PMSF, 1X PIC, pH = 8.0). SAM was omitted in the negative control reactions. For the time courses (Figs 1 and 5), a single reaction was run for each type of histone, and a 20μl aliquot was taken at each time point. Each aliquot was immediately mixed with 10μl 3X SDS-PAGE sample buffer and denatured at 100°C for 5 minutes. The zero time point was taken before SAM was added; the others were taken at the denoted number of minutes after SAM was added. A 20μl control reaction without SAM was also run, and also three tubes containing only unmodified histone H3, H3K4me3, or H3K36me3, at a concentration of 10ng/μl, in HMT buffer. Upon completion of the assay, 15μl of each time point and the negative control were loaded onto one 4–15% SDS-PAGE gel (Bio-Rad, http://www.bio-rad.com/), while 5μl of each were loaded onto another gel. 5μl of the three histone-alone samples were loaded onto both gels. The gels were run at 100V, and transferred to nitrocellulose upon completion. The 15μl blot was then probed with α-H3K36me3 (1:5000, Active Motif cat#61101, https://www.activemotif.com/) antibody, while the 5μl blot was probed with α-H3K4me3 antibody (1:5000, EMD Millipore cat#07–473, http://www.emdmillipore.com/). Less was loaded onto the H3K4me3 blot to avoid saturation of the bands due to the higher efficiency of the H3K4 trimethylation reaction. Both blots were then stripped and re-probed with α-Histone H3 antibody (1:5000, Active Motif cat#61277, https://www.activemotif.com/). The secondary antibody for all three primary antibodies was HRP-conjugated goat anti-rabbit (Bio-Rad cat#1662408EDU, http://www.bio-rad.com/), used at a concentration of 1:20,000. The experiments in Figs 1 and 5 were each run at least 3 times; a representative experiment is shown for each.

### Acid-extraction of histones

Histones were extracted from the spermatocytes of homozygous B6;129P2-*Prdm9*^tm1Ymat^/J mice, which are *Prdm9*-null [19], and C57BL/6J mice, which are wild-type for *Prdm9*. Spermatocytes were isolated and prepared into single-cell suspensions from 1–4 14dpp mice using the same previously published protocol used to prepare samples for H3K4me3 and H3K36me3 ChIP [14]. The cells were not fixed with formaldehyde after counting, however. Instead, the cells were pelleted and washed twice with 1mL ice-cold phosphate buffered saline (PBS). They were then resuspended in Triton Extraction Buffer (TEB; PBS with 0.5% Triton X-100, 1mM PMSF, and 1X PIC) at a density of 10^7^ cells/mL. Cells were lysed for 10 minutes on a rotator at 4°C, then centrifuged at 6500xg for 10 minutes at 4°C to pellet the nuclei. The supernatant was discarded and the pellet washed with half the previous volume of TEB, and centrifuged as before. The pellet was then resuspended in 0.2N HCl at a density of 4x10^7^ cells/mL (minimum volume 200μl), and incubated overnight at 4°C with gentle rotation. The following day, the cells were centrifuged at 6500xg for 10 minutes at 4°C to pellet insoluble material, and the supernatant collected. This supernatant was neutralized with 0.2 volumes of a 1M Tris solution with an unadjusted pH, which yielded a histone solution with a pH between 7 and 8 (verified with pH paper). Total protein was measured via Bradford assay, and histone solutions were stored at -80°C.

### Histone immunoprecipitation

For each immunoprecipitation, 20μl of magnetic Protein A Dynabeads (ThermoFisher, https://www.thermofisher.com/) were added to a 1.5mL microcentrifuge tube. The beads were pelleted magnetically, and the supernatant discarded. The beads were then washed 3 times with 100μl of RB buffer—RIPA buffer (50mM Tris, 150mM NaCl, 1% NP-40, 0.5% sodium deoxycholate, 0.1% SDS, pH = 8.0) with 50mg/mL BSA. After the final wash, beads were resuspended in 100μl RB, to which 10μg α-H3K4me3 antibody (EMD Millipore cat# 07–473, http://www.emdmillipore.com/) were added. The suspension was rotated for 20 minutes at room temperature to conjugate the antibody, after which the beads were pelleted magnetically and the supernatant discarded. The beads were again washed 3 times with 100μl RB, then resuspended in RB with 1mM PMSF and 1X PIC. 4μg of acid-extracted histone solution was added, to a total volume of 200μl. The no-antibody control was done by the same procedure, except no antibody was added to the beads. Immunoprecipitation was allowed to proceed overnight at 4°C with rotation. The following day, the beads were pelleted magnetically, and the supernatant discarded. The beads were then washed 3 times with 100μl RB and twice with 100μl 1X TE (10mM Tris, 1mM EDTA, pH = 8.0). During the final TE wash, the beads were transferred to a clean microcentrifuge tube. After the final wash, beads were eluted in 30μl 1X SDS-PAGE sample buffer for 10 minutes at 100°C. The beads were pelleted magnetically and the supernatant collected and immediately electrophoresed.

For the input samples, 0.5μg of histone solution was mixed with 6μl of 5X SDS-PAGE sample buffer and sufficient water to bring the volume to 30μl. The samples were mixed well and immediately denatured for 10 minutes at 100°C.

Electrophoresis was carried out at 100V, on two identical 4–15% SDS-PAGE gels (Bio-Rad, http://www.bio-rad.com/), into which 12μl of each of the IP, control, and input samples were loaded. Upon completion, the gels were transferred to nitrocellulose and immunoblotted using the SNAP id 2.0 protein detection system. One blot was probed with α-H3K4me3 antibody (1:3000, EMD Millipore cat# 07–473, http://www.emdmillipore.com/) as the primary and Veriblot for IP (1:200, Abcam cat#ab131366, http://www.abcam.com/) as the secondary. The other blot was probed with a mouse monoclonal α-H3K36me3 antibody (1:1000, Active Motif cat#61021, https://www.activemotif.com/) as the primary and an HRP-conjugated goat α-mouse antibody (1:20,000, Bio-Rad cat#170–6516, http://www.bio-rad.com/) as the secondary. After imaging, both blots were stripped and re-probed with α-Histone H3 antibody (1:3000, Active Motif cat#61277, https://www.activemotif.com/) as the primary and Veriblot for IP as the secondary. The blot in S5 Fig was also stripped and re-probed with α-Histone H4 antibody (1:1000, Active Motif cat#61521) as the primary and Veriblot for IP as the secondary.

## Supporting Information

S1 Fig Purity and activity of MBP-PRDM9 used for methylation analysis on histone peptide arrays. **(A)** Silver staining of the final prep after two-step purification on SP-sepharose and amylose beads. The ~130 kDa fraction (black arrow) was estimated to contain >85% of the protein by densitometry analysis. Molecular weight marker positions are shown on the right. **(B)** H3K4 trimethylation activity of purified MBP-PRDM9. Recombinant histone 3 was mixed with the methyl donor S-adenosylmethionine (SAM) in the absence (1) or presence (2) of purified MBP-PRDM9. A Western blot with α-H3K4me3 antibody is shown, which was subsequently stripped and re-probed with α-Histone H3 as a loading control.
(TIF)

S2 Fig Histone methylation activity of expressed and purified full-length mouse PRDM9. A histone peptide array (Active Motif cat. # 13001) containing two identical panels of 384 covalently modified N-terminal peptides from H3, H4, H2A and H2B was incubated with MBP-PRDM9 in the presence of (^3^H-methyl)-S-adenolsylmethionine and autoradiographed. **(A)** Incubation with 42 ng/μl MBP-PRDM9. **(B)** Incubation with 125 ng/μl MBP-PRDM9. Positive signals were detected with peptides representing H3 1–19 (red boxes) and H3 26–45 (blue boxes) only. Data analysis is presented in the text and in S1 Table.
(TIF)

S3 Fig Analysis of histone peptide array data. **(A)** PRDM9 substrate specificity towards H3K4 and H3K9 (H3 1–19), and H3K36 (H3 26–45). The assay for K4 methyltransferase activity was done in the presence of K9me3, and the assay for K9 methyltransferase activity in the presence of K4me3, in order to evaluate the HMT activity of PRDM9 for each individual residue. All of the H3 1–19 peptides in this assay, with the exception of K4me0 and K9me0, also contained R2me2a and R8me2a. H3 26–45 peptides were free of any additional modifications. Each bar represents two independent replicates. **(B-E)** Additional histone modifications that were found to affect PRDM9 HMT activity. Each bar represents two independent replicates. For the K4- and K9-acetylated peptides, additional individual modifications are as follows: R8me2s for K4me1, K4me2, K9me1, and K9me2; R2me2s for K4me3; R2me2a for K9me0; R8me2s for K4me0. Error bars represent standard error of the mean between two technical replicates within the same array (S2A Fig). R2me2s: dimethylated argingine 2, symmetric; R2me2a: dimethylated arginine 2, asymmetric; R8me2s: dimethylated arginine 8, symmetric; R8me2a: dimethylated arginine 8, asymmetric; T3P: phosphorylated threonine 3; S10P: phosphorylated serine 10; T11P: phosphorylated threonine 11.
(TIF)

S4 Fig H3K4me3 and H3K36me3 ChIP-seq enrichment patterns in germ cells. This figure shows a snapshot of the normalized H3K36me3 and H3K4me3 ChIP-seq data in B6 14dpp spermatocytes as visualized in the UCSC Genome Browser. The top three tracks show (1) the locations of known hotspots (B6 Hotspots), (2) ZINBA broad regions of enrichment (ZINBA Broad), and (3) ZINBA localized regions of enrichment within broad regions (ZINBA Refined). Boxed regions show zoomed-in views at promoters (red boxes), genic hotspots that show additional H3K36me3 enrichment above that associated with transcription (blue boxes), and intergenic hotspots (black boxes). In the zoomed-in views, H3K4me3 and H3K36me3 are shown on the same scale at hotspots to show the relatively weaker H3K36me3 peaks; the scales are different in the large image due to the intrinsically lower signal-to-noise ratio of H3K36me3 ChIP-seq data. Note the exclusion of H3K36me3 at promoters, compared to its coincidence with H3K4me3 at hotspots; also note the similar shapes of the H3K4me3 and H3K36me3 peaks at both genic and intergenic hotspots.
(TIF)

S5 Fig Biological replicate of α-H3K4me3 immunoprecipitation experiment. This figure shows a replicate of the experiment in Fig 6, using acid-extracted histones from different animals. This experiment was done in the same way as that in Fig 6, except instead of duplicate blots, the samples were run on a single SDS-PAGE gel and transferred to a single blot. This blot was probed with α-H3K36me3, then stripped and re-probed with α-H3K4me3, then with α-Histone H3, then with α-Histone H4. The arrow shows the faint H4 band in the *Prdm9*^+/+^ IP.
(TIF)

S6 Fig H3K4me3, H3K36me3, and DMC1 ChIP-seq enrichment in the pseudoautosomal region (PAR). This figure shows, in the C57BL/6J strain, H3K4me3, H3K36me3, and DMC1 ChIP-seq enrichment in a high-mappability segment of the PAR on the X chromosome, as visualized in the UCSC Genome Browser. Note the abrupt, substantial, and coincident increase in H3K4me3 and H3K36me3 in this PRDM9-independent region of recombination, and the concomitant increase in meiotic DSB activity as measured by DMC1 ChIP-seq. The H3K4me3 and H3K36me3 data are normalized as reads per million (RPM). The DMC1 ChIP-seq data are from Brick et al. (2012).
(TIF)

S1 Table Histone peptide array data. This table shows the positions and identities of the histone peptides on the array, and the raw signal and data analysis for each peptide. Peptides showing a positive signal are marked in red.
(XLSX)

## References

[1] Chan AH, Jenkins PA, Song YS (2012) Genome-wide fine-scale recombination rate variation in Drosophila melanogaster. PLoS Genet 8: e1003090 10.1371/journal.pgen.1003090 23284288 PMC3527307

[2] Kaur T, Rockman MV (2014) Crossover heterogeneity in the absence of hotspots in Caenorhabditis elegans. Genetics 196: 137–148. 10.1534/genetics.113.158857 24172135 PMC3872180

[3] Tischfield SE, Keeney S (2012) Scale matters: the spatial correlation of yeast meiotic DNA breaks with histone H3 trimethylation is driven largely by independent colocalization at promoters. Cell Cycle 11: 1496–1503. 10.4161/cc.19733 22433953 PMC3341227

[4] Choi K, Zhao X, Kelly KA, Venn O, Higgins JD, et al (2013) Arabidopsis meiotic crossover hot spots overlap with H2A.Z nucleosomes at gene promoters. Nat Genet 45: 1327–1336. 10.1038/ng.2766 24056716 PMC3812125

[5] Parvanov ED, Petkov PM, Paigen K (2010) Prdm9 controls activation of mammalian recombination hotspots. Science 327: 835 10.1126/science.1181495 20044538 PMC2821451

[6] Steiner CC, Ryder OA (2013) Characterization of Prdm9 in equids and sterility in mules. PLoS One 8: e61746 10.1371/journal.pone.0061746 23613924 PMC3632555

[7] Sandor C, Li W, Coppieters W, Druet T, Charlier C, et al (2012) Genetic variants in REC8, RNF212, and PRDM9 influence male recombination in cattle. PLoS Genet 8: e1002854 10.1371/journal.pgen.1002854 22844258 PMC3406008

[8] Myers S, Bowden R, Tumian A, Bontrop RE, Freeman C, et al (2010) Drive against hotspot motifs in primates implicates the PRDM9 gene in meiotic recombination. Science 327: 876–879. 10.1126/science.1182363 20044541 PMC3828505

[9] Baudat F, Buard J, Grey C, Fledel-Alon A, Ober C, et al (2010) PRDM9 is a major determinant of meiotic recombination hotspots in humans and mice. Science 327: 836–840. 10.1126/science.1183439 20044539 PMC4295902

[10] Axelsson E, Webster MT, Ratnakumar A, Consortium L, Ponting CP, et al (2012) Death of PRDM9 coincides with stabilization of the recombination landscape in the dog genome. Genome Res 22: 51–63. 10.1101/gr.124123.111 22006216 PMC3246206

[11] Sun F, Fujiwara Y, Reinholdt LG, Hu J, Saxl RL, et al (2015) Nuclear localization of PRDM9 and its role in meiotic chromatin modifications and homologous synapsis. Chromosoma 124: 397–415. 10.1007/s00412-015-0511-3 25894966 PMC4550572

[12] Grey C, Barthes P, Chauveau-Le Friec G, Langa F, Baudat F, et al (2011) Mouse PRDM9 DNA-binding specificity determines sites of histone H3 lysine 4 trimethylation for initiation of meiotic recombination. PLoS Biol 9: e1001176 10.1371/journal.pbio.1001176 22028627 PMC3196474

[13] Brick K, Smagulova F, Khil P, Camerini-Otero RD, Petukhova GV (2012) Genetic recombination is directed away from functional genomic elements in mice. Nature 485: 642–645. 10.1038/nature11089 22660327 PMC3367396

[14] Baker CL, Walker M, Kajita S, Petkov PM, Paigen K (2014) PRDM9 binding organizes hotspot nucleosomes and limits Holliday junction migration. Genome Res 24: 724–732. 10.1101/gr.170167.113 24604780 PMC4009602

[15] Romanienko PJ, Camerini-Otero RD (2000) The mouse Spo11 gene is required for meiotic chromosome synapsis. Mol Cell 6: 975–987. 10.1016/s1097-2765(00)00097-6 11106738

[16] Mahadevaiah SK, Turner JM, Baudat F, Rogakou EP, de Boer P, et al (2001) Recombinational DNA double-strand breaks in mice precede synapsis. Nat Genet 27: 271–276. 11242108 10.1038/85830

[17] Kauppi L, Jasin M, Keeney S (2013) How much is enough? Control of DNA double-strand break numbers in mouse meiosis. Cell Cycle 12: 2719–2720. 10.4161/cc.26079 23966150 PMC3899183

[18] Keeney S, Lange J, Mohibullah N (2014) Self-organization of meiotic recombination initiation: general principles and molecular pathways. Annu Rev Genet 48: 187–214. 10.1146/annurev-genet-120213-092304 25421598 PMC4291115

[19] Hayashi K, Yoshida K, Matsui Y (2005) A histone H3 methyltransferase controls epigenetic events required for meiotic prophase. Nature 438: 374–378. 16292313 10.1038/nature04112

[20] Eram MS, Bustos SP, Lima-Fernandes E, Siarheyeva A, Senisterra G, et al (2014) Trimethylation of histone H3 lysine 36 by human methyltransferase PRDM9 protein. J Biol Chem 289: 12177–12188. 10.1074/jbc.M113.523183 24634223 PMC4002121

[21] Wu H, Mathioudakis N, Diagouraga B, Dong A, Dombrovski L, et al (2013) Molecular basis for the regulation of the H3K4 methyltransferase activity of PRDM9. Cell Rep 5: 13–20. 10.1016/j.celrep.2013.08.035 24095733

[22] Koh-Stenta X, Joy J, Poulsen A, Li R, Tan Y, et al (2014) Characterization of the histone methyltransferase PRDM9 using biochemical, biophysical and chemical biology techniques. Biochem J 461: 323–334. 10.1042/BJ20140374 24785241

[23] Gu B, Lee MG (2013) Histone H3 lysine 4 methyltransferases and demethylases in self-renewal and differentiation of stem cells. Cell Biosci 3: 39 10.1186/2045-3701-3-39 24172249 PMC3953348

[24] Pfister SX, Ahrabi S, Zalmas LP, Sarkar S, Aymard F, et al (2014) SETD2-dependent histone H3K36 trimethylation is required for homologous recombination repair and genome stability. Cell Rep 7: 2006–2018. 10.1016/j.celrep.2014.05.026 24931610 PMC4074340

[25] Aymard F, Bugler B, Schmidt CK, Guillou E, Caron P, et al (2014) Transcriptionally active chromatin recruits homologous recombination at DNA double-strand breaks. Nat Struct Mol Biol 21: 366–374. 10.1038/nsmb.2796 24658350 PMC4300393

[26] Edmunds JW, Mahadevan LC, Clayton AL (2008) Dynamic histone H3 methylation during gene induction: HYPB/Setd2 mediates all H3K36 trimethylation. EMBO J 27: 406–420. 18157086 10.1038/sj.emboj.7601967 PMC2168397

[27] Rashid NU, Giresi PG, Ibrahim JG, Sun W, Lieb JD (2011) ZINBA integrates local covariates with DNA-seq data to identify broad and narrow regions of enrichment, even within amplified genomic regions. Genome Biol 12: R67 10.1186/gb-2011-12-7-r67 21787385 PMC3218829

[28] Walker M, Billings T, Baker CL, Powers N, Tian H, et al (2015) Affinity-seq detects genome-wide PRDM9 binding sites and reveals the impact of prior chromatin modifications on mammalian recombination hotspot usage. Epigenetics Chromatin 8: 31 10.1186/s13072-015-0024-6 26351520 PMC4562113

[29] Jee J, Rozowsky J, Yip KY, Lochovsky L, Bjornson R, et al (2011) ACT: aggregation and correlation toolbox for analyses of genome tracks. Bioinformatics 27: 1152–1154. 10.1093/bioinformatics/btr092 21349863 PMC3072554

[30] Yamada S, Ohta K, Yamada T (2013) Acetylated Histone H3K9 is associated with meiotic recombination hotspots, and plays a role in recombination redundantly with other factors including the H3K4 methylase Set1 in fission yeast. Nucleic Acids Res 41: 3504–3517. 10.1093/nar/gkt049 23382177 PMC3616738

[31] Cobb J, Cargile B, Handel MA (1999) Acquisition of competence to condense metaphase I chromosomes during spermatogenesis. Dev Biol 205: 49–64. 9882497 10.1006/dbio.1998.9101

[32] Sims RJ 3rd, Reinberg D (2009) Processing the H3K36me3 signature. Nat Genet 41: 270–271. 10.1038/ng0309-270 19240748

[33] Li F, Mao G, Tong D, Huang J, Gu L, et al (2013) The histone mark H3K36me3 regulates human DNA mismatch repair through its interaction with MutSalpha. Cell 153: 590–600. 10.1016/j.cell.2013.03.025 23622243 PMC3641580

[34] Pekowska A, Benoukraf T, Zacarias-Cabeza J, Belhocine M, Koch F, et al (2011) H3K4 tri-methylation provides an epigenetic signature of active enhancers. EMBO J 30: 4198–4210. 10.1038/emboj.2011.295 21847099 PMC3199384

[35] Baker CL, Kajita S, Walker M, Saxl RL, Raghupathy N, et al (2015) PRDM9 drives evolutionary erosion of hotspots in Mus musculus through haplotype-specific initiation of meiotic recombination. PLoS Genet 11: e1004916 10.1371/journal.pgen.1004916 25568937 PMC4287450

[36] Wagner EJ, Carpenter PB (2012) Understanding the language of Lys36 methylation at histone H3. Nat Rev Mol Cell Biol 13: 115–126. 10.1038/nrm3274 22266761 PMC3969746

[37] Ho TH, Park IY, Zhao H, Tong P, Champion MD, et al (2015) High-resolution profiling of histone h3 lysine 36 trimethylation in metastatic renal cell carcinoma. Oncogene. 10.1038/onc.2015.221 PMC4679725 26073078

[38] Buard J, Rivals E, Dunoyer de Segonzac D, Garres C, Caminade P, et al (2014) Diversity of Prdm9 zinc finger array in wild mice unravels new facets of the evolutionary turnover of this coding minisatellite. PLoS One 9: e85021 10.1371/journal.pone.0085021 24454780 PMC3890296

[39] Kono H, Tamura M, Osada N, Suzuki H, Abe K, et al (2014) Prdm9 polymorphism unveils mouse evolutionary tracks. DNA Res 21: 315–326. 10.1093/dnares/dst059 24449848 PMC4060951

[40] Berg IL, Neumann R, Lam KW, Sarbajna S, Odenthal-Hesse L, et al (2010) PRDM9 variation strongly influences recombination hot-spot activity and meiotic instability in humans. Nat Genet 42: 859–863. 10.1038/ng.658 20818382 PMC3092422

[41] Ponting CP (2011) What are the genomic drivers of the rapid evolution of PRDM9? Trends Genet 27: 165–171. 10.1016/j.tig.2011.02.001 21388701

[42] Lieber MR (2010) The mechanism of double-strand DNA break repair by the nonhomologous DNA end-joining pathway. Annu Rev Biochem 79: 181–211. 10.1146/annurev.biochem.052308.093131 20192759 PMC3079308

