# Supplementary figures and images for: The Meiotic Recombination Activator PRDM9 Trimethylates Both H3K36 and H3K4 at Recombination Hotspots In Vivo
Source: PLoS Genet. 2016 Jun 30;12(6):e1006146. doi: 10.1371/journal.pgen.1006146 (PMC4928815; doi:10.1371/journal.pgen.1006146)

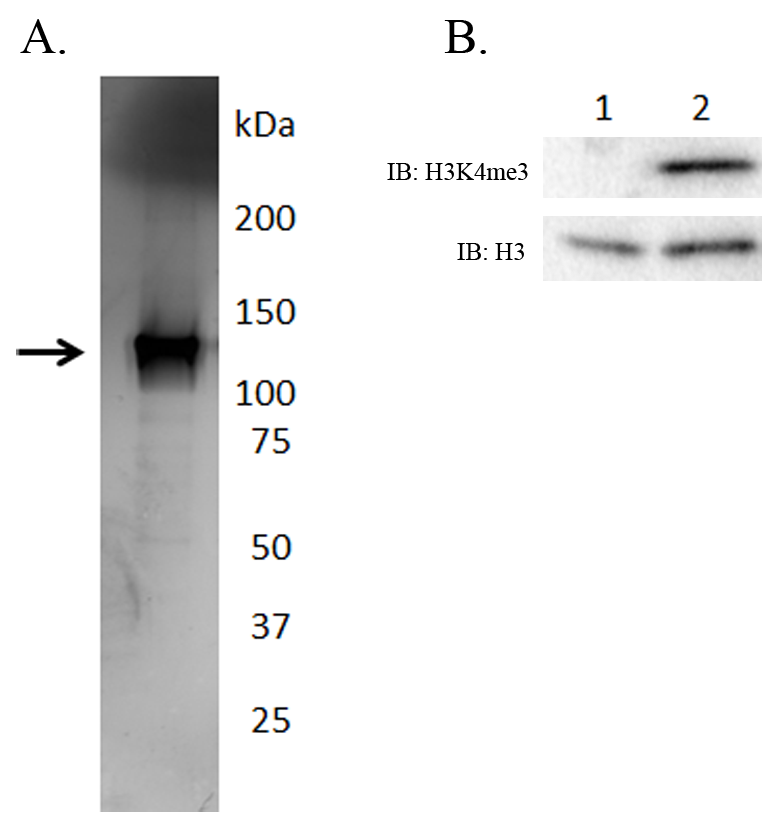

Supplement: S1 Fig — (A) Silver staining of the final prep after two-step purification on SP-sepharose and amylose beads. The ~130 kDa fraction (black arrow) was estimated to contain >85% of the protein by densitometry analysis. Molecular weight marker positions are shown on the right. (B) H3K4 trimethylation activity of purified MBP-PRDM9. Recombinant histone 3 was mixed with the methyl donor S-adenosylmethionine (SAM) in the absence (1) or presence (2) of purified MBP-PRDM9. A Western blot with α-H3K4me3 antibody is shown, which was subsequently stripped and re-probed with α-Histone H3 as a loading control. (TIF) [file pgen.1006146.s001.tif]

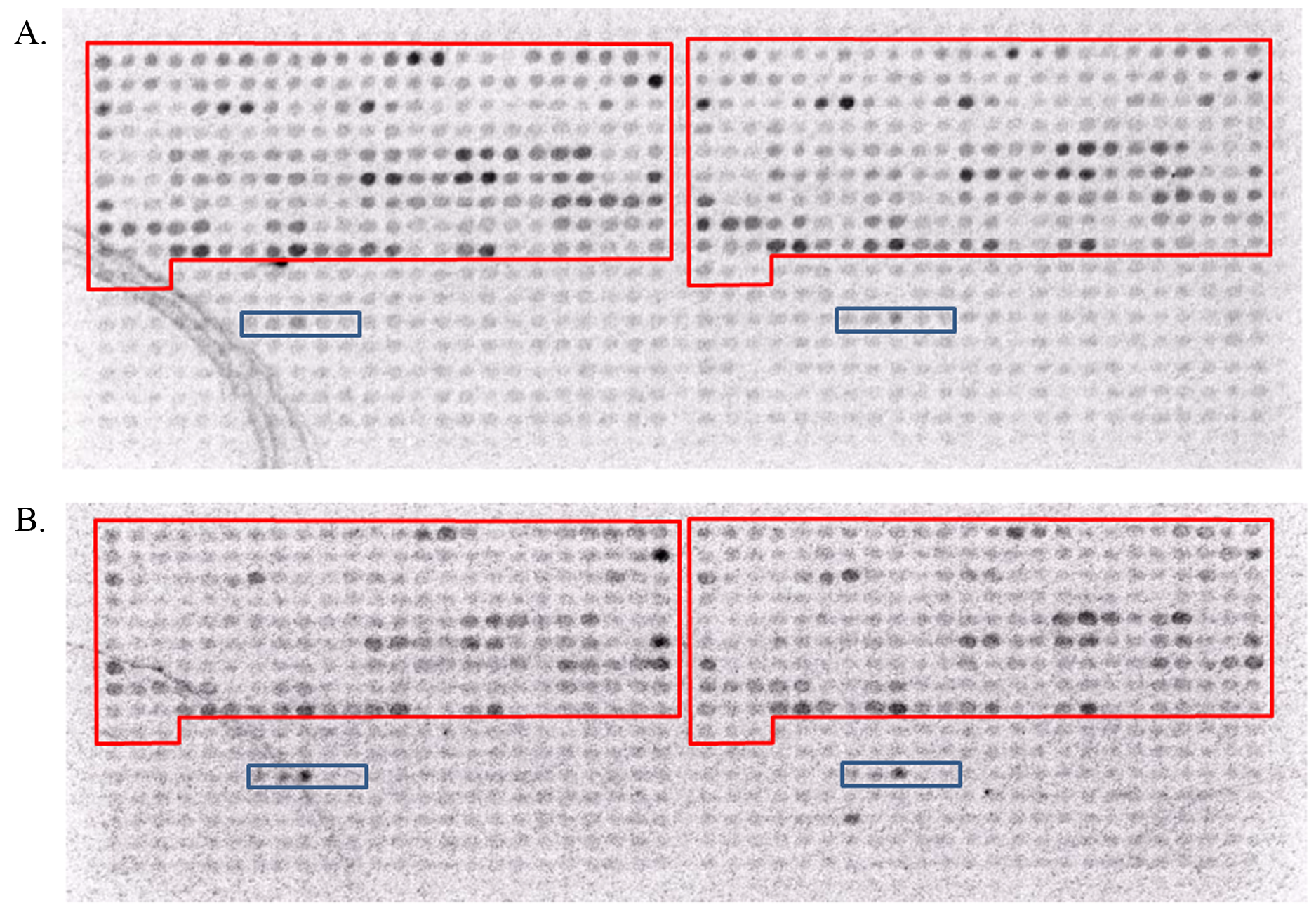

Supplement: S2 Fig — A histone peptide array (Active Motif cat. # 13001) containing two identical panels of 384 covalently modified N-terminal peptides from H3, H4, H2A and H2B was incubated with MBP-PRDM9 in the presence of (3H-methyl)-S-adenolsylmethionine and autoradiographed. (A) Incubation with 42 ng/μl MBP-PRDM9. (B) Incubation with 125 ng/μl MBP-PRDM9. Positive signals were detected with peptides representing H3 1–19 (red boxes) and H3 26–45 (blue boxes) only. Data analysis is presented in the text and in S1 Table. (TIF) [file pgen.1006146.s002.tif]

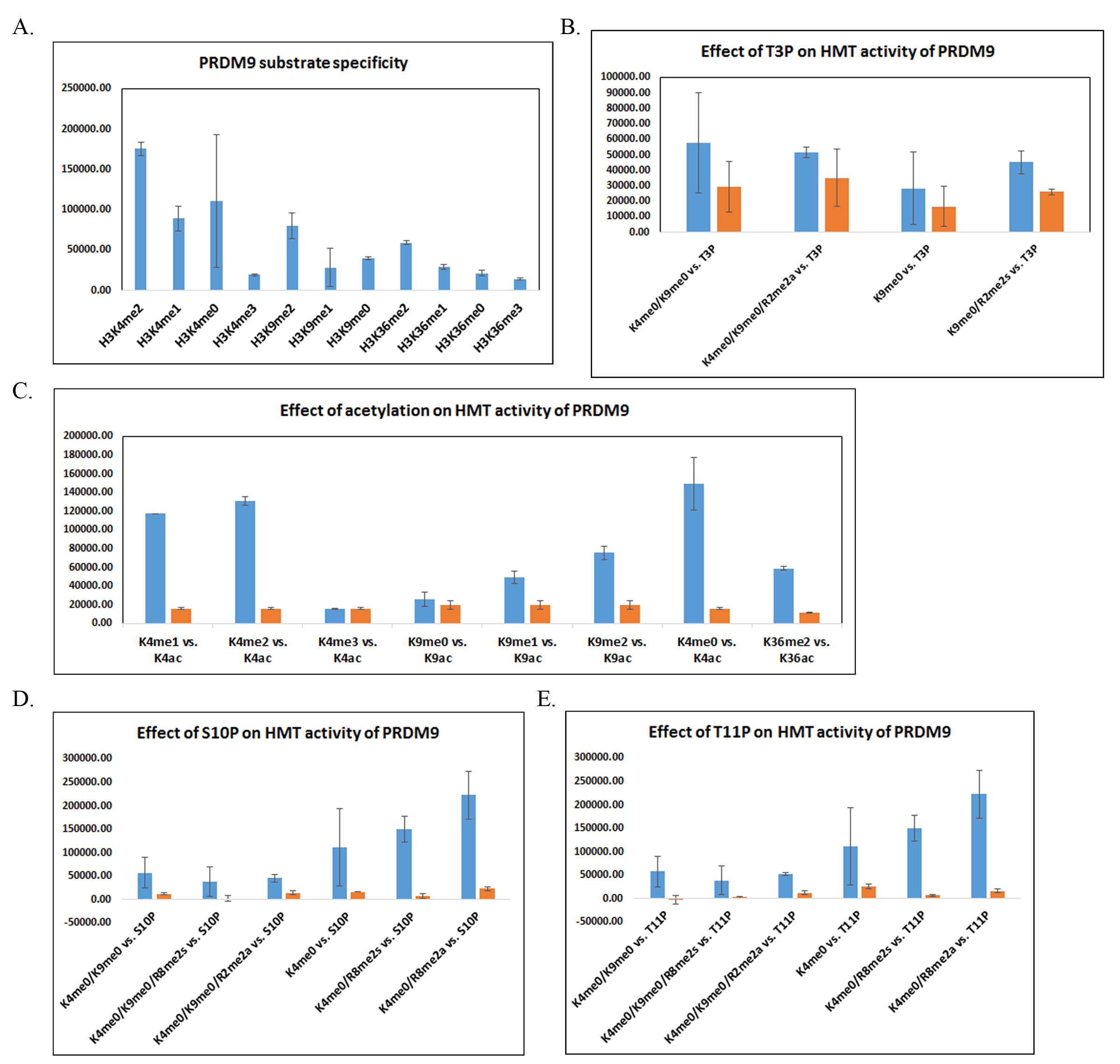

Supplement: S3 Fig — (A) PRDM9 substrate specificity towards H3K4 and H3K9 (H3 1–19), and H3K36 (H3 26–45). The assay for K4 methyltransferase activity was done in the presence of K9me3, and the assay for K9 methyltransferase activity in the presence of K4me3, in order to evaluate the HMT activity of PRDM9 for each individual residue. All of the H3 1–19 peptides in this assay, with the exception of K4me0 and K9me0, also contained R2me2a and R8me2a. H3 26–45 peptides were free of any additional modifications. Each bar represents two independent replicates. (B-E) Additional histone modifications that were found to affect PRDM9 HMT activity. Each bar represents two independent replicates. For the K4- and K9-acetylated peptides, additional individual modifications are as follows: R8me2s for K4me1, K4me2, K9me1, and K9me2; R2me2s for K4me3; R2me2a for K9me0; R8me2s for K4me0. Error bars represent standard error of the mean between two technical replicates within the same array (S2A Fig). R2me2s: dimethylated argingine 2, symmetric; R2me2a: dimethylated arginine 2, asymmetric; R8me2s: dimethylated arginine 8, symmetric; R8me2a: dimethylated arginine 8, asymmetric; T3P: phosphorylated threonine 3; S10P: phosphorylated serine 10; T11P: phosphorylated threonine 11. (TIF) [file pgen.1006146.s003.tif]

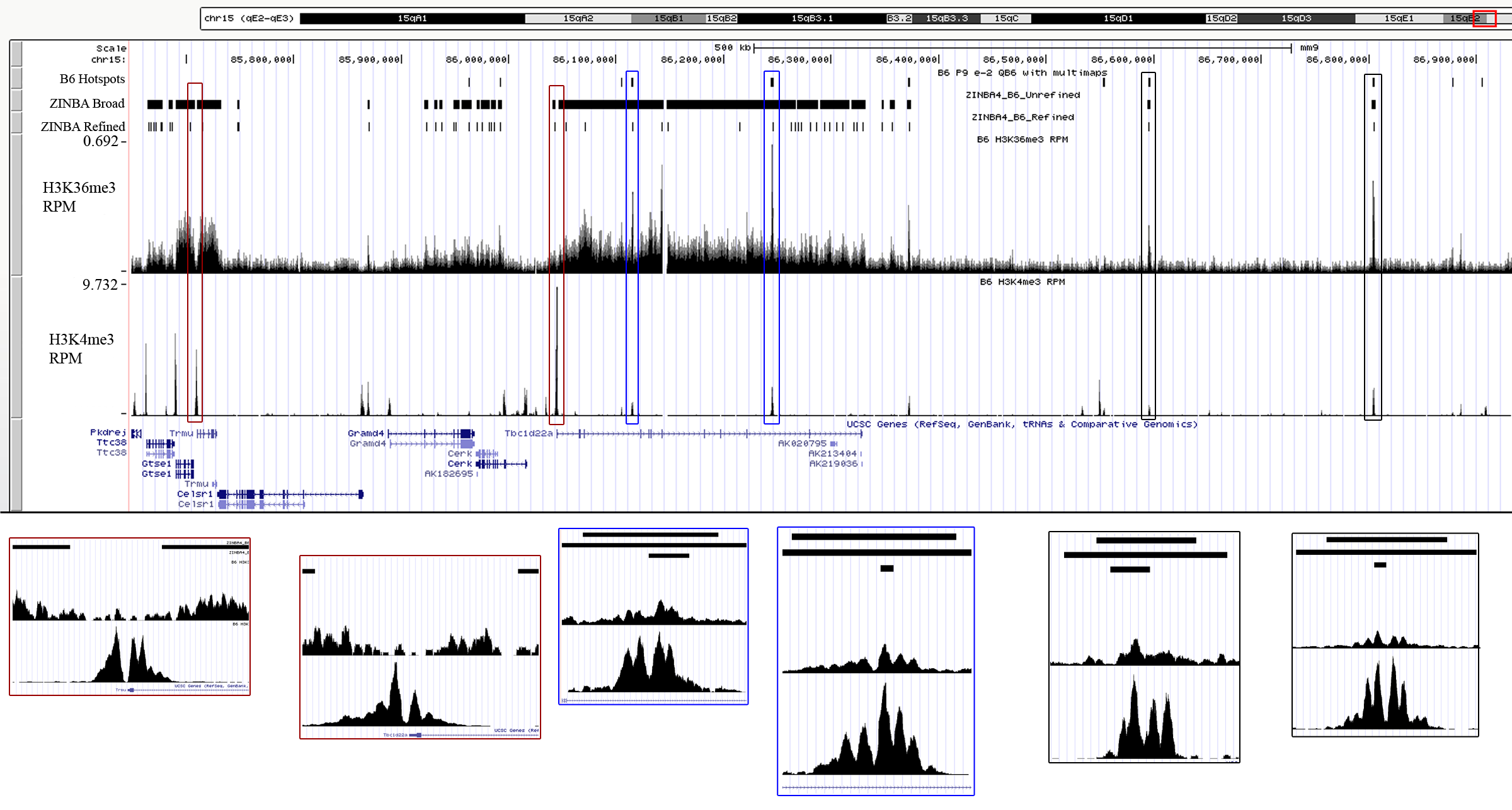

Supplement: S4 Fig — This figure shows a snapshot of the normalized H3K36me3 and H3K4me3 ChIP-seq data in B6 14dpp spermatocytes as visualized in the UCSC Genome Browser. The top three tracks show (1) the locations of known hotspots (B6 Hotspots), (2) ZINBA broad regions of enrichment (ZINBA Broad), and (3) ZINBA localized regions of enrichment within broad regions (ZINBA Refined). Boxed regions show zoomed-in views at promoters (red boxes), genic hotspots that show additional H3K36me3 enrichment above that associated with transcription (blue boxes), and intergenic hotspots (black boxes). In the zoomed-in views, H3K4me3 and H3K36me3 are shown on the same scale at hotspots to show the relatively weaker H3K36me3 peaks; the scales are different in the large image due to the intrinsically lower signal-to-noise ratio of H3K36me3 ChIP-seq data. Note the exclusion of H3K36me3 at promoters, compared to its coincidence with H3K4me3 at hotspots; also note the similar shapes of the H3K4me3 and H3K36me3 peaks at both genic and intergenic hotspots. (TIF) [file pgen.1006146.s004.tif]

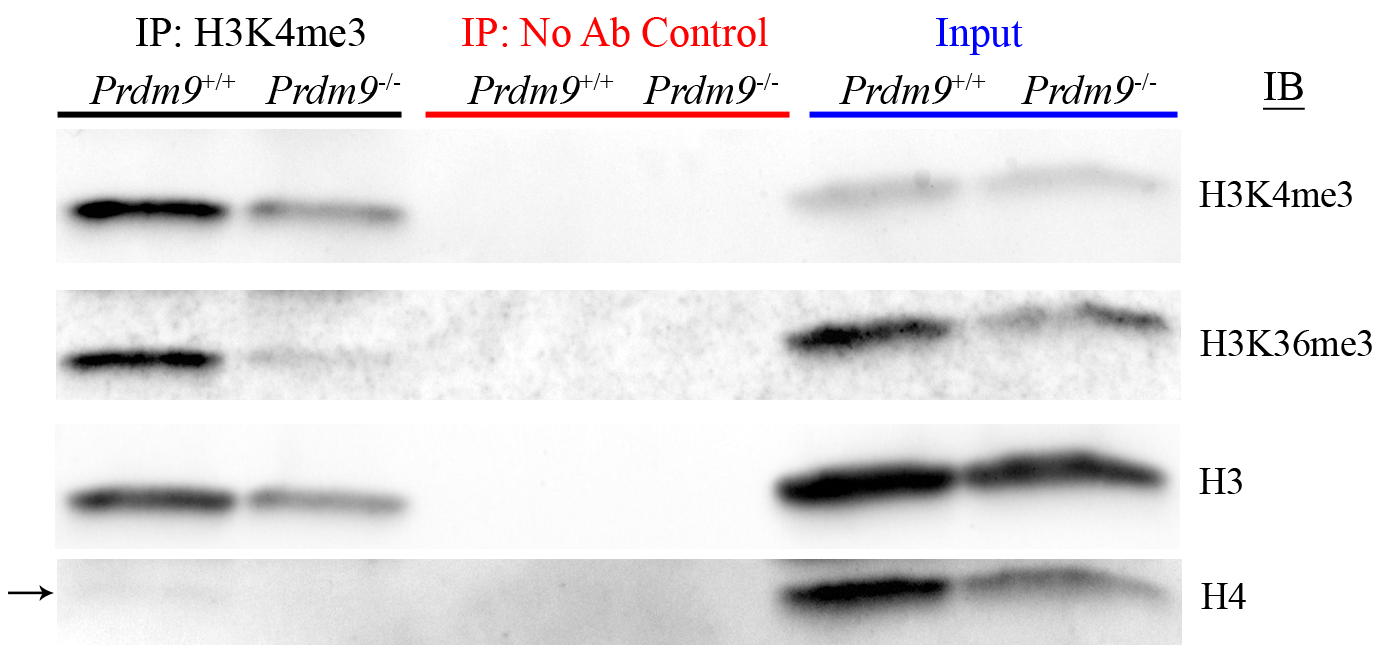

Supplement: S5 Fig — This figure shows a replicate of the experiment in Fig 6, using acid-extracted histones from different animals. This experiment was done in the same way as that in Fig 6, except instead of duplicate blots, the samples were run on a single SDS-PAGE gel and transferred to a single blot. This blot was probed with α-H3K36me3, then stripped and re-probed with α-H3K4me3, then with α-Histone H3, then with α-Histone H4. The arrow shows the faint H4 band in the Prdm9+/+ IP. (TIF) [file pgen.1006146.s005.tif]

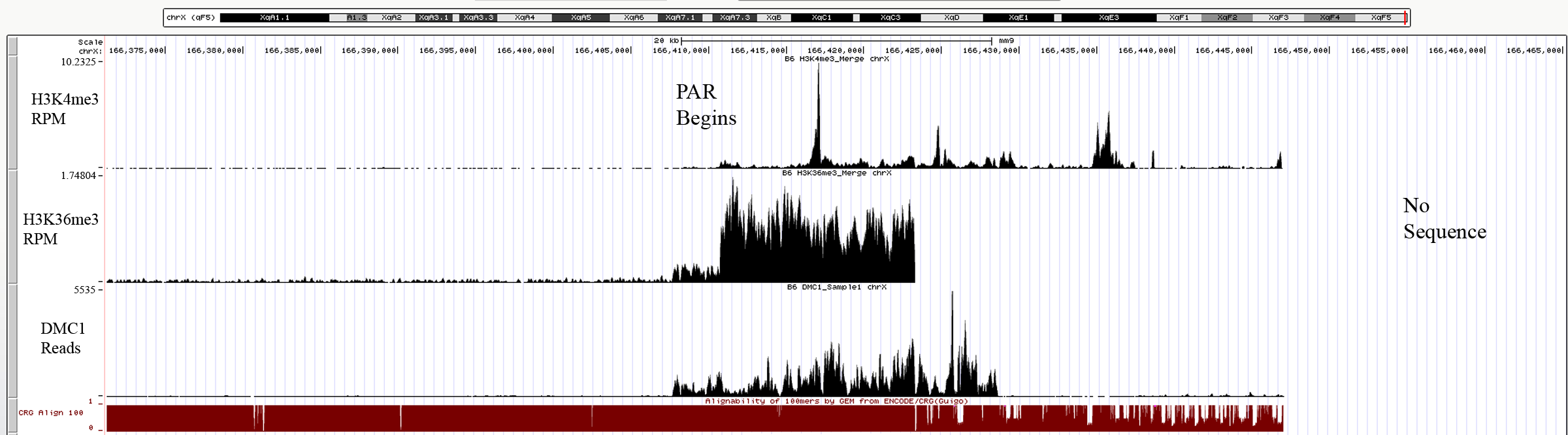

Supplement: S6 Fig — This figure shows, in the C57BL/6J strain, H3K4me3, H3K36me3, and DMC1 ChIP-seq enrichment in a high-mappability segment of the PAR on the X chromosome, as visualized in the UCSC Genome Browser. Note the abrupt, substantial, and coincident increase in H3K4me3 and H3K36me3 in this PRDM9-independent region of recombination, and the concomitant increase in meiotic DSB activity as measured by DMC1 ChIP-seq. The H3K4me3 and H3K36me3 data are normalized as reads per million (RPM). The DMC1 ChIP-seq data are from Brick et al. (2012). (TIF) [file pgen.1006146.s006.tif]
